# MicroRNA Responses Associated with Vector-Borne Diseases in Humans and Arthropod Vectors: A Systematic Review

**DOI:** 10.3390/ijms27198444

**Published:** 2026-09-22

**Authors:** Tilini K. De Silva, John Stenos, Stephen R. Graves, Tarka R. Bhatta, Nathan B. Unsworth, Ryan J. Farr, Cameron R. Stewart, Simon M. Firestone

**Affiliations:** 1 Department of Veterinary Biosciences, Melbourne Veterinary School, Faculty of Science, The University of Melbourne, Parkville, VIC 3052, Australia; tilinikausha@student.unimelb.edu.au; 2 Australian Rickettsial Reference Laboratory, University Hospital, Geelong, VIC 3220, Australia; john.stenos@barwonhealth.org.au (J.S.); graves.rickettsia@gmail.com (S.R.G.); tarka.bhatta@barwonhealth.org.au (T.R.B.); 3 Sensors and Effectors Division, Defence Science and Technology Group, 506 Lorimer Street, Fisherman’s Bend, Melbourne, VIC 3207, Australia; nathan.unsworth1@defence.gov.au; 4 CSIRO Health & Biosecurity, Australian Centre for Disease Preparedness, Geelong, VIC 3220, Australia; ryan.farr@csiro.au (R.J.F.); cameron.stewart@csiro.au (C.R.S.)

**Keywords:** biomarkers, differential expression, microRNA, miRNAs, mosquitoes, systematic review, ticks, vector-borne diseases, vectors

## Abstract

Vector-borne diseases (VBDs) represent a significant public health challenge. Early and accurate diagnosis is crucial for effective disease management and control, with host-encoded microRNAs (miRNAs) emerging as promising disease biomarkers. This systematic review surveys research conducted on miRNA responses in disease vectors and human hosts of VBDs. A systematic search of four databases and manual searching identified 93 qualifying articles. Numerous VBDs were described, with the most common focus on dengue (21%; *n* = 15) and Chagas disease (11%; *n* = 8). Twenty-eight in vivo human studies have characterised miRNAs linked to VBDs, including African trypanosomiasis, Chagas disease, Crimean-Congo haemorrhagic fever, dengue, Japanese encephalitis, leishmaniasis, malaria, onchocerciasis, schistosomiasis, and Zika virus disease. Some overlaps of miRNA responses to infection were observed for Chagas disease (miR-208a), dengue (miR-146-5p), and schistosomiasis (sma-miR-bantam), while the majority exhibited minimal overlap. Studies addressing miRNA profiling of arthropod vectors predominantly emphasise ticks, with many efforts focusing on the miRNA profiles of tick saliva, salivary glands, nymphal ticks, and adults due to their pivotal role in pathogen transmission. This review reports a paucity of studies investigating miRNA biomarkers for VBD diagnosis and prognosis in humans and nominates the development of standardised protocols and validation across study cohorts as future directions.

## 1. Introduction

VBDs are a major cause of illness worldwide, particularly in tropical and subtropical areas [1]. The economic and societal ramifications of VBDs are multifaceted, incorporating high expenditures related to healthcare, with global costs estimated at US $12 billion annually, largely characterised by declines in productivity [2], control and prevention expenses [3], social disruption [4] and disproportionate effects on socioeconomically challenged populations [5].

The diagnosis of VBDs is often challenging. Serological cross-reactivity between flaviviruses, such as dengue virus serotypes, Zika virus, West Nile virus, and Japanese Encephalitis virus (JEV), occurs due to the structural similarities shared by the envelope proteins of these flaviviruses, leading to the production of antibodies that can bind to antigens from multiple viruses within the same family [6]. Current diagnostics frequently lack sensitivity and specificity during the early stages of infection. In the case of malaria, rapid diagnostic tests (RDTs), including lateral flow immunochromatographic antigen detection assays, often demonstrate inadequate sensitivity in detecting early-stage infections with low parasite densities [7]. Likewise, conventional microscopy methods for diagnosing leishmaniasis, although widely employed, demonstrate limited sensitivity, particularly at low parasite burdens [8]. Similarly, in Lyme disease, the antibody levels are often undetectable within the first two to four weeks post-exposure [9].

The shortcomings of established diagnostic techniques have prompted investigation of alternative strategies to improve VBD detection. MicroRNAs (miRNAs) are a class of small (18–22 nucleotide) non-coding RNAs that play a crucial role in post-transcriptional gene regulation [10]. MicroRNAs function by binding to complementary regions on target messenger RNAs (mRNAs), which results in mRNA degradation or translational repression [11].

MiRNAs offer promise as non-invasive biomarkers for several reasons. They have high accessibility in bodily fluids such as blood, serum, plasma, and urine [12], allowing for minimally invasive sample collection. Additionally, these miRNAs show a certain degree of stability in biofluids, primarily since they are protected from degradation by their association with protein complexes or encapsulation in extracellular vesicles [13]. The early response of miRNAs to infections is intrinsically linked to the host’s pathogen detection mechanisms, notably including pattern recognition receptors (PRRs), which identify pathogen-associated molecular patterns (PAMPs) and subsequently activate immune responses [14]. Thereby, in the context of infection, specific miRNAs are differentially expressed, contributing to the precise modulation of the immune response [15]. Many studies have identified early miRNA responses to human infections, including COVID-19 [16], hepatitis [17,18,19], human immunodeficiency virus/ acquired immunodeficiency syndrome (HIV/AIDS) [20], and tuberculosis [21,22].

Though traditional diagnostic methods (PCR, microscopy, serology, antigen detection) have established roles in infectious illness diagnosis, miRNAs offer potential advantages, especially during specific clinical windows. Antigen-based and microscopy methods often exhibit reduced sensitivity during early infection when pathogen burden is low [23]; conversely, miRNAs are rapidly dysregulated in response to PAMPs and may possess the potential to detect infection before conventional markers [14]. While being highly sensitive, PCR-based diagnostics rely on the presence of pathogen-derived nucleic acids and cannot reliably distinguish between persistent infection and active replication [24]; in contrast, miRNA responses could potentially stratify infection status (acute vs. chronic), may reflect the host’s dynamic immune responses [25,26], and could indicate disease severity and prognosis by capturing immune dysregulation before clinical manifestations emerge [27]. Similarly, serology generally cannot differentiate persistent from active infections, or even just past exposure, without paired sera [28]; miRNA profiles could reflect disease stages over time [27]. Additionally, miRNA responses in VBD systems are altered in response to pathogen infection and may influence vector competence for pathogen transmission [29]. However, although miRNA research in VBDs has expanded considerably in recent years, these diagnostic and prognostic applications remain largely exploratory and require rigorous clinical validation before widespread implementation [27].

Despite significant advancements in miRNA biomarker research, knowledge remains limited regarding their role in VBD detection and diagnosis. This systematic review comprehensively assesses current evidence on the use of miRNAs as biomarkers for VBDs in humans and vector species. Limitations, challenges, and future directions for integrating miRNA-based diagnostic techniques into practice are also discussed.

## 2. Materials and Methods

### 2.1. Review Protocol

This systematic review was registered with the International Prospective Register of Systematic Reviews (PROSPERO, ID: CRD42023460764) [30] to ensure transparency, and the reporting follows the ‘Preferred Reporting Items for Systematic Reviews and Meta-Analyses’ (PRISMA) guidelines (https://www.prisma-statement.org, accessed on 20 May 2026). The PRISMA checklist is provided in the Supplementary Materials, which outlines the comprehensive reporting of this review (Supplementary Tables S1 and S2).

### 2.2. Search Strategy

A search strategy was initially developed in Web of Science. Four electronic databases (Web of Science (all databases), CAB Abstracts, Scopus, and PubMed) were queried for peer-reviewed articles published in English from 2003 to 2025 (Phase 1). Additional articles were identified by utilising email alerts for recently published literature from various databases.

Following peer review feedback, the search strategy was expanded (Phase 2) to include additional vector-borne disease vectors to ensure exhaustive coverage of the literature. Both search phases employed identical Boolean operators and methodology adapted to each database’s syntax requirements. Complete search strings, filters, and search dates for both phases are provided in Supplementary Table S3.

Manual searches were conducted to identify additional studies not captured by electronic database searches. This included screening reference lists of included studies (backward citation searching), identifying studies citing relevant review articles (forward citation tracking), and conducting supplementary keyword searches in Google Scholar, with multiple pages of results screened for eligible studies.

The utilisation of the PICO (Population-Intervention-Comparison-Outcome) framework was implemented to devise the search strategy, adapted from elsewhere [31]. Within the scope of this study, the “Populations” of interest were human and vector populations exhibiting distinct miRNA expression patterns attributed to VBD exposure. As per the “Intervention” of the review framework, we reviewed papers on miRNA profiling of human patients associated with VBDs, arthropod vectors associated with VBDs, and relevant cell culture studies. Given the comprehensive assessment of all miRNA expressions associated with VBDs, no specific “Comparison” was integrated into the search strategy.

### 2.3. Quality Assessment and Selection

All search results were imported into EndNote 21.2 (Clarivate Analytics, Philadelphia, PA, USA) for initial organisation. Records were then transferred to Covidence (https://www.covidence.org, Veritas Health Innovation, Melbourne, Australia) for deduplication and title and abstract screening. Duplicate records were removed using Covidence’s automated deduplication function. Subsequently, all articles underwent screening based on their title and abstract using the following exclusion criteria: (1) Studies detecting miRNAs in hosts other than humans and vector species; (2) Studies detecting miRNAs associated with diseases other than VBDs; (3) Studies not having any data on miRNA expression levels; (4) Studies not having miRNA differential expression levels; (5) Studies not mentioning the specific up/down regulation of miRNAs; (6) Studies not published in English; (7) Conference abstracts, editorials, reviews, books or book chapters, reports, theses, dissertations, blogs, newsletters, and non-peer-reviewed sources. For mosquito-related studies identified in Phase 2, a publication year filter was applied during title and abstract screening, limiting inclusion to publications from 2018 onwards, as a comprehensive review published in 2018 has already covered this topic [32]. Following that, full-text articles were obtained for chosen references via the University of Melbourne’s library and through inter-library loan services. Selected articles were then fully read to assess their eligibility for inclusion. Studies presenting data on differential miRNA expression attributed to VBDs in humans and vectors were included (see Figure 1).

### 2.4. Data Extraction and Analyses

Data extraction was carried out by TKDS, and any inconsistencies were addressed through deliberation with fellow authors, who served as secondary reviewers. The following relevant information from qualifying studies was extracted and recorded in an Excel spreadsheet: host type (human, vector), vector species, location of sample, sample/control characteristics, vector sample type (egg, larvae, nymph, adult cell type used for cell-culture studies, expression level/pattern of differentially expressed miRNAs (significantly upregulated and downregulated), VBD type, and the geographic region of VBDs. These data were then combined, and any discrepancies were discussed and agreed upon by the authors. Owing to the wide range of study designs examined, there was no one critical appraisal instrument that could be applied; rather, a narrative critical comparison of the pertinent study characteristics was generated.

## 3. Results

### 3.1. Study Characteristics

#### 3.1.1. Literature Search and Eligible Articles

For the Phase 1 search, a total of 935 studies were identified across the four databases. After removing duplicates, 427 studies underwent title and abstract screening. Thirty-five additional articles were found through hand searching for full-text review. A total of 105 articles met the inclusion criteria and were evaluated in full text. For the Phase 2 search, a total of 1105 studies were identified across the same four databases. After removing duplicates, 446 studies underwent title and abstract screening. A total of 45 articles met the inclusion criteria and were evaluated in full text. After the full-text evaluation from both searches, a total of 93 studies were included in the review (Figure 1).

#### 3.1.2. General Characteristics of Studies Included in the Review

The host species covered by the review include human (53%), tick (21%), mosquito (18%), other (cells used in vitro studies: 4%), tsetse fly (3%), lice (<1%), and mite (<1%) (Figure 2). Focus VBDs include African trypanosomiasis, anaplasmosis, babesiosis, Chagas disease, chikungunya virus disease, Crimean-Congo haemorrhagic fever, dengue, filariasis, JE, leishmaniasis, Lyme disease, malaria, Mayaro virus disease, Onchocerciasis, O’nyong-nyong virus disease, Powassan virus disease, rickettsia, schistosomiasis, severe fever with thrombocytopenia syndrome, tick-borne encephalitis virus, West Nile virus disease and Zika virus disease. The most common VBDs were dengue (21%), Chagas disease (11%), and malaria (9%) (Figure 2). Most studies were conducted during the period 2013–2023 (Figure 3).

#### 3.1.3. Geographic Distribution of Studies Included in the Review

Of the 93 studies presenting data on differential miRNA expression attributed to VBDs in humans and vectors, by region, the most patient/vector samples originated from Asia (*n* = 20), followed by Africa (*n* = 6), South America (*n* = 6), and North America (*n* = 3) (Figure 4). Most studies were conducted on humans (*n* = 49), followed by ticks (*n* = 15), predominantly from China (*n* = 12) (Figure 5). The studies involving humans as the host were undertaken in Argentina, Bolivia, Brazil, Cameroon, Chile, China, Colombia, Ghana, Guinea, India, Iraq, Italy, Mozambique, Singapore, Spain, Thailand, Turkey, Zimbabwe and Uganda (Figure 5).

### 3.2. Differential miRNA Expression in Patients Associated with Vector-Borne Diseases

Twenty-eight studies investigated in vivo human tissue components (whole blood, serum, plasma, exosomes, cerebrospinal fluid exosomes, and heart tissue) to detect differential miRNAs associated with VBDs, including African trypanosomiasis, Chagas Disease (American trypanosomiasis), Crimean-Congo haemorrhagic fever, dengue, JEV disease, leishmaniasis, malaria, onchocerciasis, schistosomiasis, and Zika virus disease (Table 1). The sample size among these 28 studies varies from 8 to 204 patients, while the proportions of males and females range from 25% to 89% and 11% to 75%, respectively. Studies on African trypanosomiasis and schistosomiasis have been conducted in Africa, and on American trypanosomiasis (also known as “Chagas disease”) in South America, given the disease endemicity (Table 1). More extensive research has been conducted on miRNA responses to VBDs in vitro. Chagas disease, dengue, JE, and leishmaniasis are the only four VBDs for which both in vivo and in vitro data are available (Table 2).

Changes in miRNA signature due to human African trypanosomiasis, which is caused by the parasite *Trypanosoma brucei gambiense*, and transmitted to humans by bites of tsetse flies (*Glossina* spp.), has been in vivo (sample type: whole blood) investigated in a study conducted in Guinea, in which miR-193b has been reported to be significantly upregulated, and miR-119a-3p, miR-27b, miR-126, miR-98, miR-409-3p, miR-4291, miR-146b-5p, and miR-454 were significantly downregulated in both stage I (initial hemolymphatic stage) and stage II cases (late meningo-encephalitic stage involving the central nervous system [83] of trypanosomiasis [33]. In this study, three individual miRNAs: miR-199a-3p, miR-27b and miR-126 were able to differentiate trypanosomiasis patients from controls [33]. Moreover, three studies have conducted in vivo miRNA profiling of human heart tissue, plasma and exosomes from plasma and serum from patients infected with Chagas disease (also known as American trypanosomiasis), which is caused by the parasite *Trypanosoma cruzi*, which is transmitted by triatomine bugs (Order Hemiptera: Family Reduviidae) [84]. Interestingly, in many studies, the cases with “chronic Chagas disease cardiomyopathy” (CCC) showed an overlap in the expression of miR-208a [35,36,38,40] In addition, miR-208a was observed to play a role in fibrosis and cardiac dysfunction in Chagas disease [38]. The convergent identification of miR-208a across multiple CCC studies represents a biologically replicated finding with potential diagnostic value, warranting clinical validation to establish its utility as a cardiac biomarker in Chagas disease. Furthermore, there was a notable overlap in the expression of miR-21-5p across studies conducted both in vivo and in vitro, where it was significantly upregulated in all CCC samples of plasma/serum exosomes, cardiac tissue, and human cardiac fibroblasts [40]. However, these findings require cross-study validation using standardised protocols and consistent analytical methods before clinical translation.

Although there were no overlaps of differentially expressed miRNAs in the four malaria in vivo studies, each study has identified several miRNAs as potential biomarkers for distinguishing between various forms of malaria infection. miR-150-5p and miR-15b-5p were identified as potential biomarkers for *P. vivax* malaria, while let-7a-5p was identified for detecting both *P. vivax* and *P. falciparum* infections; however, these biomarkers showed reduced diagnostic sensitivity and specificity [45] than the existing diagnostic techniques, including RDTs, microscopy and molecular detection (PCR) [85]. Moreover, in another study, RT-qPCR analysis demonstrated that miR-7977, miR-28-3p, miR-378a-5p, miR-194-5p, and miR-3667-5p were significantly upregulated in patients with complicated *P. vivax* infection compared to those with uncomplicated infection (according to the WHO-based criteria for severe malaria patients [86]) and the diagnostic potential of these miRNAs was also evaluated and found to be significant, with favourable sensitivity and specificity [44]. Furthermore, another study has identified plasma miR-451 and miR-16 as potential biomarkers for malaria pathogenesis, in which it has revealed a statistically significant downregulation of the expression of both these miRNAs in *P. vivax* infections relative to uninfected control groups [42]. Additionally, a separate study focusing on children with diverse clinical manifestations of malaria identified miR-4497 as a promising miRNA candidate for distinguishing severe malaria [43]. In summary, even though the malaria studies have suggested several panels of miRNAs as potential biomarkers to detect different types of malaria on their own, the absence of consistent miRNA overlap across these investigations suggests that further research using standardised protocols and miRNA detection techniques is required.

In vivo schistosomiasis studies have identified several parasite-derived miRNAs from the serum and extracellular vesicles of infected patients, in which sma-miR-bantam was significantly upregulated in both studies and in both serum and extracellular vesicles [48,49]. One study reported that miRNA biomarkers offer advantages over conventional serology for evaluating clearance of infection and detecting secondary exposure to schistosomiasis [49]. Although serological tests yield a higher sensitivity for detecting IgG antibodies due to *Schistosoma* spp. infection, the results remain positive indefinitely after infection and cannot distinguish active infections from past exposure or monitor treatment efficacy [49]. Therefore, they have detected *S. mansoni*-derived sma-miR-bantam and sma-miR-2c-3p with a sensitivity of 80–86% and a specificity of 84–94%, with significantly lower levels of expression in post-treatment patients compared to pre-treatment patients, and with complete disappearance of expression in some cured patients [49]. The other study on schistosomiasis has identified *S. mansoni*-derived sma-miR-277 and sma-miR-3479-3p as promising biomarkers, achieving sensitivity and specificity of 80–90% and 80–89%, respectively [48]. Furthermore, miR-223 has been identified as a potential biomarker for human schistosomiasis in a separate study [47].

VBDs of viral aetiology have garnered the most attention in studies examining miRNA expression profiles in vivo (Table 2). In Crimean-Congo haemorrhagic fever, a total of 198, 192 and 205 miRNAs were expressed in whole blood of controls, cases and fatal cases, respectively, including the significant upregulation of five, 10, and 15 miRNAs, and significant downregulation of 10, 66, and 52 miRNAs in non-fatal cases vs. controls, fatal vs. controls, and non-fatal vs. fatal cases, respectively (Table 2) [51]. Samples from six fatal cases of CCHF showed unique miRNA signatures, with upregulation of miR-4793-3p, miR-195-3p, miR-4440, and miR-1909-5p, and downregulation of several let-7 family miRNAs [51]. No samples were available from severe cases before death, limiting the ability to identify miRNA patterns predictive of severe outcomes leading to death.

Several overlaps of dysregulated miRNAs have been recorded in in vivo dengue studies, including miR-146a-5p, which has been significantly downregulated in dengue, severe/ acute dengue, and dengue with a warning sign patients (platelet count, mean ± SD, ×10^3^/µL: 29 ± 5 and 3-5 days of fever) vs. controls [52,54,55]. Furthermore, a panel of miRNAs, including miR-21-5p, miR-22-5p, and miR-340-5p, has been reported to be significantly upregulated in dengue patients vs. controls [54] and to be significantly downregulated in severe dengue vs. controls, dengue with a warning sign patients, and uncomplicated dengue patients [55]. Notably, miR-320a was significantly upregulated, and miR-584-5p was significantly downregulated in severe dengue patients [55], while both miRNAs were recorded to be differentially expressed in all dengue patients within two weeks of infection [57]. miR-320a was also observed in several dengue virus-related in vitro studies [55,75]. miR-21-5p and miR-146a-5p have been identified as capable of distinguishing between dengue-infected patients (*n* = 40) and healthy controls (*n* = 32) with AUC = 0.90, 95% CI [0.83–0.97] and AUC = 0.74, 95% CI [0.63–0.86], respectively [54]. Similarly, miR-320a-5p and miR-486-5p distinguished dengue-positive patients (*n* = 26) from other febrile illness controls (*n* = 7) with AUC = 0.79, 95% CI [0.61–0.99] and AUC = 0.75, 95% CI [0.56–0.95], respectively, whilst within dengue-positive groups, these miRNAs together with miR-122-5p further distinguished uncomplicated dengue (*n* = 14) from severe dengue (*n* = 12) patients with AUC = 0.82, 95% CI [0.63–1.00]; AUC = 0.74, 95% CI [0.55–0.93]; and AUC = 0.79, 95% CI [0.62–0.96], respectively, collectively suggesting moderately high diagnostic accuracy across multiple comparisons [55]. Overlaps in differentially expressed miRNAs were also noted between in vivo and in vitro studies of dengue infection. Significant upregulation of miR-122-5p was observed in severe dengue patients and in dengue virus-infected Huh7, A549, and THP1 cells [55]. It is worth noting that miR-21-5p was observed to be significantly downregulated in dengue virus-infected Huh7 and HEK293T cells [74] and significantly upregulated in dengue patients compared to controls [54]. Additionally, miR-320c was observed to be significantly upregulated in both dengue virus-infected peripheral blood mononuclear cells [76] and also in severe dengue patients [55]. Nevertheless, these overlapping findings should be interpreted cautiously given the heterogeneity in study design, sample types, disease stages, and analytical methods across studies (Table 2), and harmonised validation studies are needed to establish these miRNAs as reliable clinical biomarkers.

Two studies have investigated microRNAs responsive to JE infection in vivo, one in blood and one in cerebrospinal fluid-derived exosomes. Discrimination between Japanese encephalitis/acute encephalitis syndrome (JE/AES) patients (*n* = 20) and non-JE/AES controls (*n* = 12) was achieved with miR-21-5p and miR-150-5p, yielding AUC = 0.78, 95% CI [0.59–0.97] and AUC = 0.81, 95% CI [0.62–1.01], respectively [59]. In addition, miR-21 was found to be upregulated, and miR-155 was downregulated in JE patients compared to healthy controls, whereas both miRNAs were recorded to be significantly upregulated in non-JE acute encephalitis syndrome cases relative to both JE-infected individuals and controls [58].

Differential expression of several miRNAs was observed to be shared among different stages of the same infection. miR-21 and miR-155 were shared among JE cases and non-JE acute encephalitis syndrome cases [58]; miR-4792 was shared between one day post-infection and two days post-infection of human astrocytes with *Borrelia burgdorferi* (strain B31 MI-16) infection [61]; and miR-4497 was shared among malaria cases, severe anaemia-prostration cases, acute respiratory distress cases, and cases confirmed with prostration, multiple seizures, and acute respiratory distress [43].

### 3.3. Differential miRNA Expression in Disease Vectors

As mentioned previously, the review entails studies that were based on disease vectors, including lice, mites, mosquitoes, ticks, and tsetse flies. Out of those, mosquito host-based studies and the respective miRNA signatures have been covered recently and comprehensively by Feng et al. [32].

#### 3.3.1. Ticks

Differentially expressed miRNAs involved in the tick life cycle from egg to adult are illustrated in Figure 6. The miRNA hlo-miR-1 is shared among all the life stages. The data on the distinct miRNA expression profiles of tick organs, including the ovary, midgut, Malpighian tubules, salivary glands, and tick saliva, are presented in Figure 7. Most of the studies were based on miRNA profiling of *Haemaphysalis longicornis,* in which many miRNAs were found to be significantly up/down regulated. Most of the studies on tick vectors have focused on the miRNA profiles of tick saliva and salivary glands since they play a crucial role in the spread of pathogenic microbes to vertebrate hosts. References to the articles on tick vectors and their differentially expressed miRNA profiles are included in the Supplementary Table S5.

#### 3.3.2. Lice, Mites, Tsetse Flies

Differentially expressed miRNAs involved in human body lice, mites and tsetse flies have also been included in the Supplementary Table S5. Differentially expressed miRNAs identified in various other in vitro host cell models are included in the Supplementary Table S6.

### 3.4. Differential miRNA Expression in Vectors Associated with Vector-Borne Diseases

The majority of in vivo and in vitro studies examining differential miRNA expression in vectors associated with VBDs concentrated on mosquito hosts, which accounted for 67% of research. Tick hosts represented the second most studied group at 22%, whilst tsetse fly hosts comprised 11% of investigations. Among mosquito species, researchers focused primarily on *Aedes aegypti*, *Aedes albopictus*, *Anopheles coluzzi*, *Anopheles gambiae*, *and Anopheles stephensi*. Tick-based studies predominantly utilised *Ixodes scapularis*, whilst tsetse fly research was limited to *Glossina morsitans morsitans* (Table 3).

For *Aedes aegypti*, a major vector for several arboviruses, differential miRNA expression was observed in response to various viral infections. In the case of chikungunya virus disease, several of the most commonly differentially expressed miRNAs were identified in different studies; however, no shared miRNAs were observed among these studies, indicating limited reproducibility of discovery findings across independent investigations [87,105,106]. Zika virus (ZIKV) infection in *Aedes aegypti* resulted in complex miRNA expression patterns that varied depending on the tissue type and days post-infection (dpi), several miRNAs, including miR-210, miR-279, miR-286b, miR-2941, miR-2946, miR-308-3p, miR-309a, miR-932-5p, miR-957, and miR-989, demonstrated biological replication across different tissue types and dpi for ZIKV infected *A. aegypti* samples [88,89] (Table 3).

*Aedes albopictus*, the major vector for chikungunya virus, dengue virus and dirofilariasis, showed differential miRNA expression in saliva with mir-125, mir-263a, mir-8, mir-184, and mir-100 in response to chikungunya virus disease [106]. Dengue virus (DENV-2) investigations in *Aedes albopictus* revealed substantial variation in miRNA profiles across experimental models. A C6/36 cell line study identified 99 known differentially expressed miRNAs, including miR-927, miR-989, and miR-34 [94], whilst temporal profiling of adult female midgut tissues identified a more selective response with let-7f, miR-1260, miR-34-3p, and miR-989 consistently dysregulated across 5, 7, and 10 dpi [95]. Notably, miR-989 and miR-34 were identified across both cell line and in vivo contexts, potentially representing biologically replicated miRNA responses to dengue infection, whilst further studies are needed to validate the extensive cell line miRNA signatures in field-collected *A. albopictus* populations to confirm their ecological relevance. Comparative studies between *Aedes aegypti* and *Aedes albopictus* revealed common differentially expressed miRNAs in response to Dengue virus (DENV-2) and Zika virus infections, including miR-23a-3p, miR-27a-3p, miR-128-3p, miR-143-3p, miR-146a-3p, miR-150-3p, miR378a-3p, and miR-423-3p [92] (Table 3).

Adult female mosquitoes of *Anopheles coluzzi* showed differential expression of one miRNA: aga-chr2L_23370 due to primary infection with O’nyong-nyong virus [96]. This represents a discovery finding that would benefit from replication across additional *Anopheles* species to establish its broader applicability as an important miRNA response to O’nyong-nyong virus infection. In *Anopheles gambiae*, several miRNAs were found to be differentially expressed in response to *Plasmodium falciparum* and *Plasmodium berghei* infections, which cause malaria. The most common differentially expressed miRNAs included aga-miR-8-3p, aga-miR-34-5p, aga-miR-989-3p, aga-miR-14-3p, aga-miR-277-3p, and aga-miR-305-5p [97]. Furthermore, when adult female mosquitoes of *Anopheles stephensi* were infected with Mayaro virus, several novel miRNAs (as-mirNOV10, as-mirNOV16, as-mirNOV17) and known miRNAs (aga-miR-286b, aga-miR-2944a, aga-miR-2944b, aga-miR-307, and aga-miR-309) were reported to be differentially expressed [98] (Table 3). The identification of novel miRNAs presents an intriguing discovery; however, independent technical validation and functional characterisation across multiple vector populations would be necessary to elucidate their biological significance in Mayaro virus pathogenesis.

In ticks, *Ixodes scapularis* salivary glands infected with Powassan virus (POWV) were reported to show statistically significant upregulation of mir-X1b, miR-1, miR-315, miR-5307, and miR-79 and statistically significant downregulation of miR-210, miR-12, miR-317, and miR-5312 [99]. Contrarily in the same species, several known miRNAs were reported to be statistically significantly upregulated (miR-87, miR-71, miR-5307, miR-2001, miR-5310, miR-317: (*p* < 0.05)) and statistically significantly downregulated (miR-79, miR-1, miR-153,miR-9a-5p, miR-1-3p: (*p* < 0.05)) due to Lyme disease causing bacterium, *Borrelia burgdorferi* infection in salivary glands and midgut infection [100]. Notably, miR-5307 and miR-317 were differentially expressed in both Powassan virus and *B. burgdorferi* infections [99,100], suggesting these may represent biologically replicated biomarkers of tick immune activation; however, this cross-pathogen replication requires independent validation. There were two studies reporting miRNA signatures in *I. scapularis* ticks due to *Anaplasma phagocytophilum* infection, which causes anaplasmosis, but no miRNAs were observed to be shared between the two studies, indicating limited reproducibility of discovery findings and underscoring the importance of standardised methodologies in tick miRNA profiling. [101,102] (Table 3).

## 4. Discussion

This systematic review examines the existing literature on the utilisation of miRNA biomarkers for the detection of VBDs in humans, the presence of miRNAs in various vector species, and identifies and contextually summarises the miRNAs that exhibit differential expression in relation to these diseases. The majority of research originated from the tropics and sub-tropics, possibly because of the favourable climates for vector survival [107] and reproduction [108], unplanned urbanisation [109], and poor access to health care [110]. Furthermore, the geographic distribution of the included studies revealed a concentration of research activity in Asia, with China making major contributions. This regional emphasis may result from the high prevalence of VBDs in these areas, which fuels the demand for advanced diagnostic techniques [111,112,113,114].

Notably, a thorough assessment of the biological origin and functional relevance of the reported miRNA responses is necessary for their interpretation. Differentially expressed miRNAs may demonstrate non-specific indicators of tissue damage or systemic inflammation, disease-specific pathogenic processes, or broad correlates of ongoing infection without direct causative involvement [12,115,116]. In many viral diseases, for instance, miRNAs associated with innate immune activation, like miR-146a, are frequently dysregulated, suggesting that they may represent more general immune responses rather than disease-specific indicators [117]. On the other hand, certain miRNAs have higher diagnostic specificity due to pathogen-specific expression patterns that arise from direct pathogen modulation of host gene expression [118]. Distinguishing between these categories is essential for clinical application, as biomarkers of systemic inflammation may lack specificity for VBD diagnosis, whereas truly disease-specific miRNAs could enable targeted therapeutic interventions [117].

Notably, there were no overlaps of differentially expressed miRNAs in the four malaria in vivo studies. This is substantially attributable to pre-analytical and analytical variability, which represents a critical barrier to biomarker standardisation and clinical implementation [119]. Reasons for this may have been the analytical variability stemming from the different miRNA profiling techniques, including RT-qPCR [42,45], RNA sequencing [43], and microarray analysis [44] and different sample types, including plasma [42,43], whole blood [44], and plasma extracellular vesicles [45]. Another confounding factor could be varying sample sizes, ranging from 19 to 204 patients (Table 1). Furthermore, the reported comparison groups across each study were also different, as some studies have undertaken a case–control comparison approach [44,45,50] while some have compared the differential miRNA expression between specific symptoms/ conditions of malaria [42,43] (Table 2). Normalisation strategies represent another critical source of variability, including the use of different endogenous controls and variable normalisation methods which could influence the reported fold-change values and statistical significance [120]. Several other pre-analytical factors could also influence miRNA quantification, which could substantially mask disease-associated miRNA signatures [121], including haemolysis (as red blood cell lysis releases abundant miRNAs [122]), storage conditions (storage duration and temperature could substantially alter miRNA integrity [123]), and RNA extraction protocols (which could yield variable miRNA quantities [124]).

Most of the studies included in the review suggest that the recent research on differential expression of miRNAs associated with vectors and VBDs has concentrated on mosquito-borne diseases, including JE, malaria, dengue, chikungunya virus disease, and Zika virus infection. This focus is driven by the global health burden these diseases impose, particularly in tropical and subtropical regions where they are endemic [125]. Numerous studies have used mosquito vectors and/or cell culture (in vitro) techniques, making use of mosquito cell lines in their sample and experimental strategy, possibly because they provide an easily attainable and controlled environment for studying the interactions between viruses and their vectors.

In terms of flaviviruses, dengue has been more extensively studied, possibly due to its significant global public health burden and widespread endemic distribution, whilst JE and Zika virus show more limited profiles. The heterogeneity in sample types employed across different flavivirus studies, including serum, plasma, cerebrospinal fluid, and cultured cells, hampers the standardisation and comparative interpretation of miRNA signatures. Furthermore, dengue serotype-specific miRNA signatures remain largely unexplored. Whilst individual studies have examined DENV-1, -2, and -3 infections, direct comparative analyses between serotypes are absent from the current literature. This represents a critical gap, as serotype-specific miRNA biomarkers could enhance diagnostic specificity and inform serotype-dependent clinical management strategies. Malaria-associated miRNA dysregulation reflects complex interactions between *Plasmodium* parasites and host immune responses, yet the specificity of reported miRNA signatures remains uncertain. Several miRNA responses were reported as having biomarker potential for detecting malaria: miR-150-5p and miR-15b-5p for *P. vivax* malaria [45], let-7a-5p for both *P. vivax* and *P. falciparum* infections [45], miR-451 and miR-16 for malaria pathogenesis [42], miR-4497 to distinguish severe malaria [43]. Despite these promising findings, the translational utility of these miRNA signatures is constrained by a lack of reproducible validation across independent studies. Whilst numerous differentially expressed miRNA responses have been reported in Chagas disease, the most noteworthy finding is the consistent identification of miR-208a across multiple CCC studies, representing a biologically replicated result with promising diagnostic value [35,36,38,40]. The consistent upregulation of the sma-miR-bantam across multiple studies [48,49] strengthens its diagnostic candidacy for schistosomiasis, whilst its significantly reduced expression following treatment highlights a key advantage over conventional serology.

Across mosquito vector studies, miR-989 emerged as the most consistently replicated miRNA, identified in both *Aedes albopictus* and *A. aegypti* across multiple infection contexts [88,89,94,95], suggesting biological robustness. However, the broader miRNA signatures reported across chikungunya, Zika, dengue, and malaria vector studies largely lack cross-study reproducibility, necessitating further independent validation. miRNAs are significant modulators of tick physiology, affecting the regulation of gene expression at the post-transcriptional level, including development [126], blood-feeding [127], and pathogen transmission [128]. MiRNA profiles from different species of ticks that are important vectors of infectious diseases, including *Haemaphysalis longicornis*, *Hyalomma anatolicum anatolicum*, *Hyalomma asiaticum*, *Ixodes ricinus*, *Rhipicephalus microplus*, and *Rhipicephalus sanguineus*, were contrastingly analysed in the review while differentiating the highly/differentially expressed miRNAs according to various developmental stages, tissues, and organs (Figure 6 and Figure 7). Most of the studies have focused on miRNA profiling of nymphal and adult ticks as opposed to other life stages, as these stages are the most responsible for disease transmission to humans [129]. Similarly, many studies have focused on miRNA profiling of tick saliva and salivary glands given their critical role in the transmission of pathogens [130,131,132,133]. Across tick vector studies, miR-5307 and miR-317 represent the most notable cross-pathogen replicated finding in *Ixodes scapularis*, differentially expressed across both Powassan virus and *Borrelia burgdorferi* infections [99,100]; however, the absence of shared miRNAs between independent *Anaplasma phagocytophilum* studies highlights the limited reproducibility characterising tick miRNA research.

Further studies are required to investigate the robustness of miRNAs as biomarkers for VBDs, as we report here only a small number of published studies in most instances. Inconsistencies were also observed in data reporting across studies (i.e., fold change values not being reported alongside statistical significance). While statistical significance may highlight important differences, fold changes indicate the magnitude of these differences [134]. When judging the actual biological relevance of such findings, statistical significance should not be considered alone; further consideration is required of the magnitude of effect, sample size, variability, uncertainty, bias, and alternative explanations for the data [135]. The reviewed studies, with sample sizes ranging from 16 to 204 patients, highlight substantial variability that may compromise result validity; smaller cohorts increase risks of false positives due to exaggerated effect sizes and false negatives from insufficient power to detect true associations [136]. Increased sample sizes enhance the precision of biomarker discovery by diminishing random variability and facilitating the identification of clinically important differences, which is particularly crucial due to the subtle alterations in miRNA expression associated with vector-borne pathogen interactions [137]. Furthermore, another common limitation observed across the reviewed studies was the underreporting of sensitivity and specificity values, alongside AUC values of the panels of biomarkers identified. This often stems from the fact that many of these studies are in the discovery phase, depicted by small patient cohorts and a lack of independent validation sets. Without a defined threshold for classification and validation on independent samples, it becomes challenging to report fixed sensitivity and specificity values [136,138].

Validation of findings described in this review requires further research, considering age, gender, genetics, nutrition, and environmental conditions [139]. For example, investigating whether miRNA responses to diseases such as malaria remain consistent across regions and demographics is crucial, as studies show variability influenced by geographic differences (this systematic review revealed that the four malaria studies from diverse regions included in the analysis exhibited minimal overlap in differentially expressed miRNAs), and genetic factors (e.g., miRNA-146a polymorphisms in Indian populations has revealed no association with malaria susceptibility, contrasting earlier findings in African cohorts [140]). Affordability of miRNA-based disease biomarkers is pertinent, especially in low-income nations most affected by VBDs [141]. The translation of novel diagnostic technologies requires a substantial market presence to attract corporate investment, considering the financial resources necessary for thorough research, validation, and regulatory approval [118]. This review is subject to some limitations. Some studies may not have been included in the review due to the restriction to English-language publications and possible missed studies despite the comprehensive search strategy. Methodological limitations included single-reviewer screening, lack of formal quality assessment, and inability to perform meta-analysis due to heterogeneity.

Therefore, the next logical step involves further investigations across diverse cohorts with large sample sizes, with statisticians assuming a pivotal role in informing trial sizes and guaranteeing robust data analysis [142]. In the future, artificial intelligence (AI) and machine learning (ML) algorithms could also be integrated to enhance VBD diagnosis through miRNA biomarkers, contingent upon the availability of new data over time [143]. Such comprehensive studies would not solely augment the reliability of miRNA biomarkers but also facilitate their incorporation into clinical practice, ultimately enhancing the diagnosis and management of VBDs [144].

## 5. Conclusions

The diversity of circulating microRNAs underscores their multifaceted potential as biomarkers for numerous vector-borne diseases, illustrating the intricacy of host–pathogen interactions and disease mechanisms. The most frequently reported differentially expressed miRNAs regarding different VBDS in different hosts and vectors were recognised through this review, potentially allowing for differentiation between pathogens, and these could theoretically be used as a foundation for future biomarker research in this area. Furthermore, although there are a few studies which have demonstrated the promising nature of potential miRNA biomarkers for VBDs, this systematic review explains the lack of research conducted in this field, especially utilising human populations. Substantial methodological and translational challenges and limitations remain in standardising and validating miRNA diagnostic methods. Therefore, forthcoming research efforts should aim at the optimisation of detection methodologies, the application of standardised protocols, and the diagnostic validation of miRNA-based assays.

## Figures and Tables

**Figure 1:**
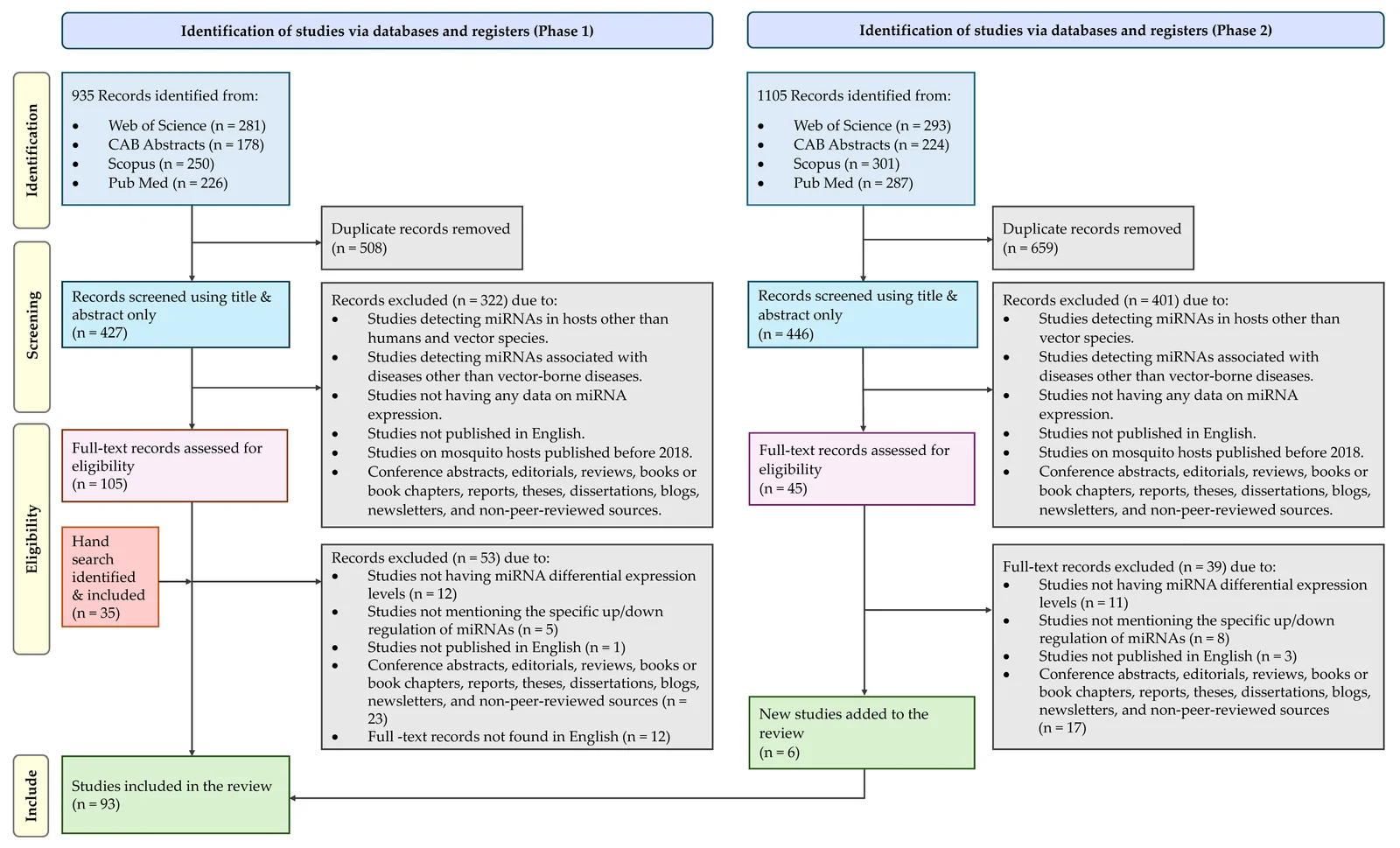
The flow diagram of Preferred Reporting Items for Systematic Reviews and Meta-Analyses (PRISMA) displays the number of articles at various stages along with the exclusion criteria applied in the present review. (Note: Following peer review, the database search (Phase 2) expanded the search strategy to ensure exhaustive coverage of the literature).

**Figure 2:**
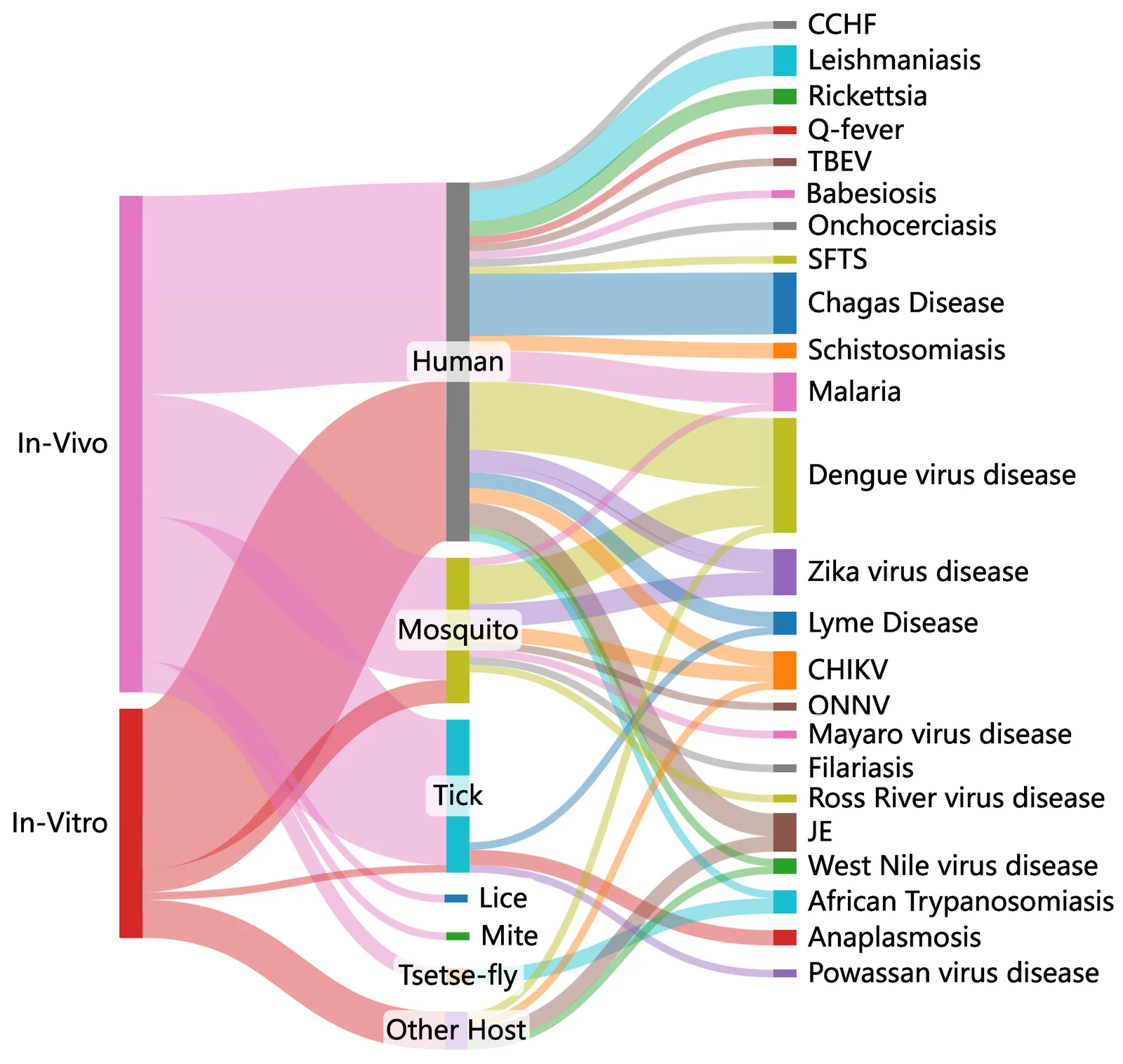
The association of study types, host types and VBDs of studies included in the review. (CCHF: Crimean-Congo haemorrhagic fever, TBEV: Tick-borne encephalitis virus, SFTS: Severe fever with thrombocytopenia syndrome, CHIKV: Chikungunya virus, JE: Japanese encephalitis, ONNV: O’nyong-nyong virus).

**Figure 3:**
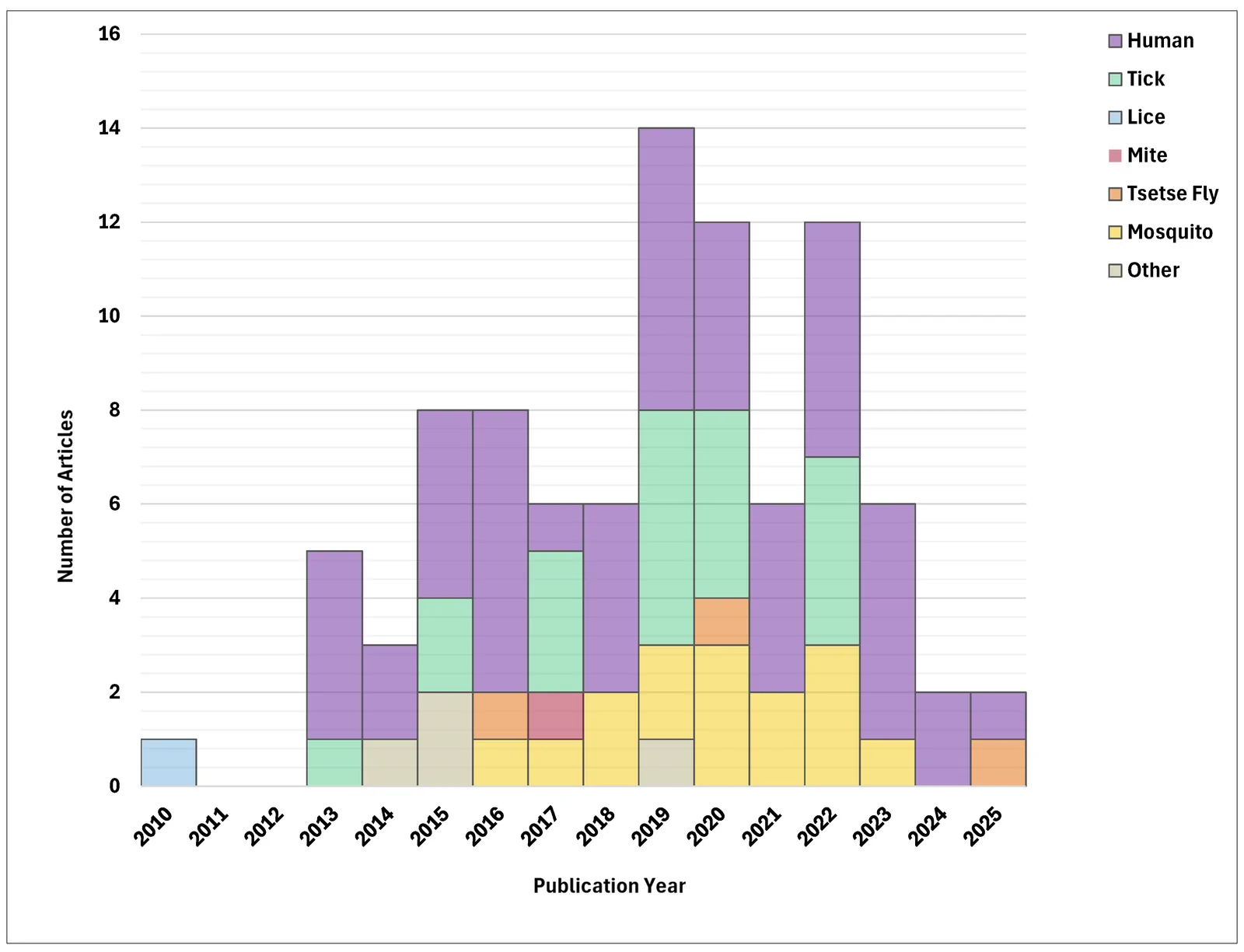
Year-wise distribution of the number of published articles selected in this systematic review according to host type.

**Figure 4:**
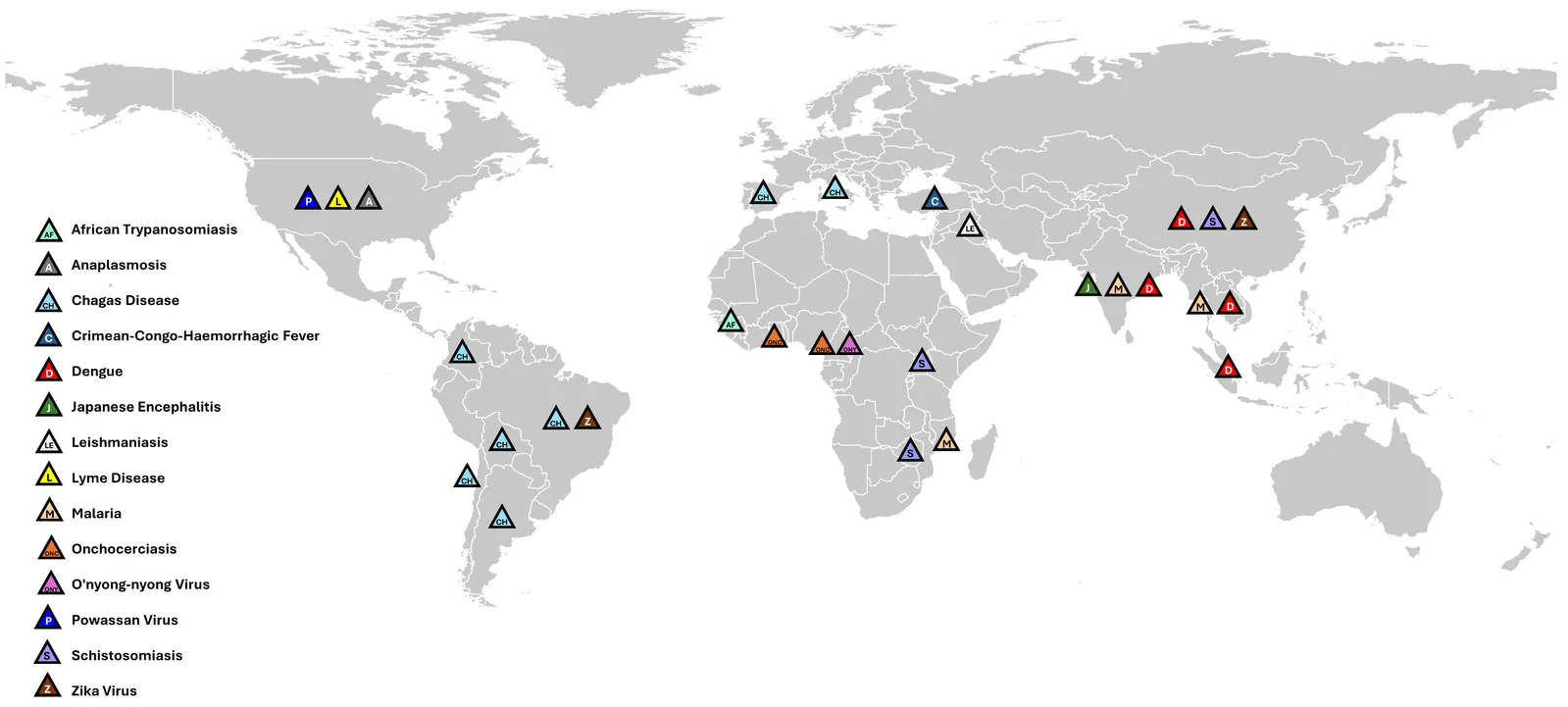
Geographical distribution of the reviewed studies according to the vector-borne disease type. (Note: Geographic representation is limited to in vivo studies that employed host-derived samples with documented collection sites. In vitro studies utilising artificially cultured cell lines or tissues were excluded from geographic representation).

**Figure 5:**
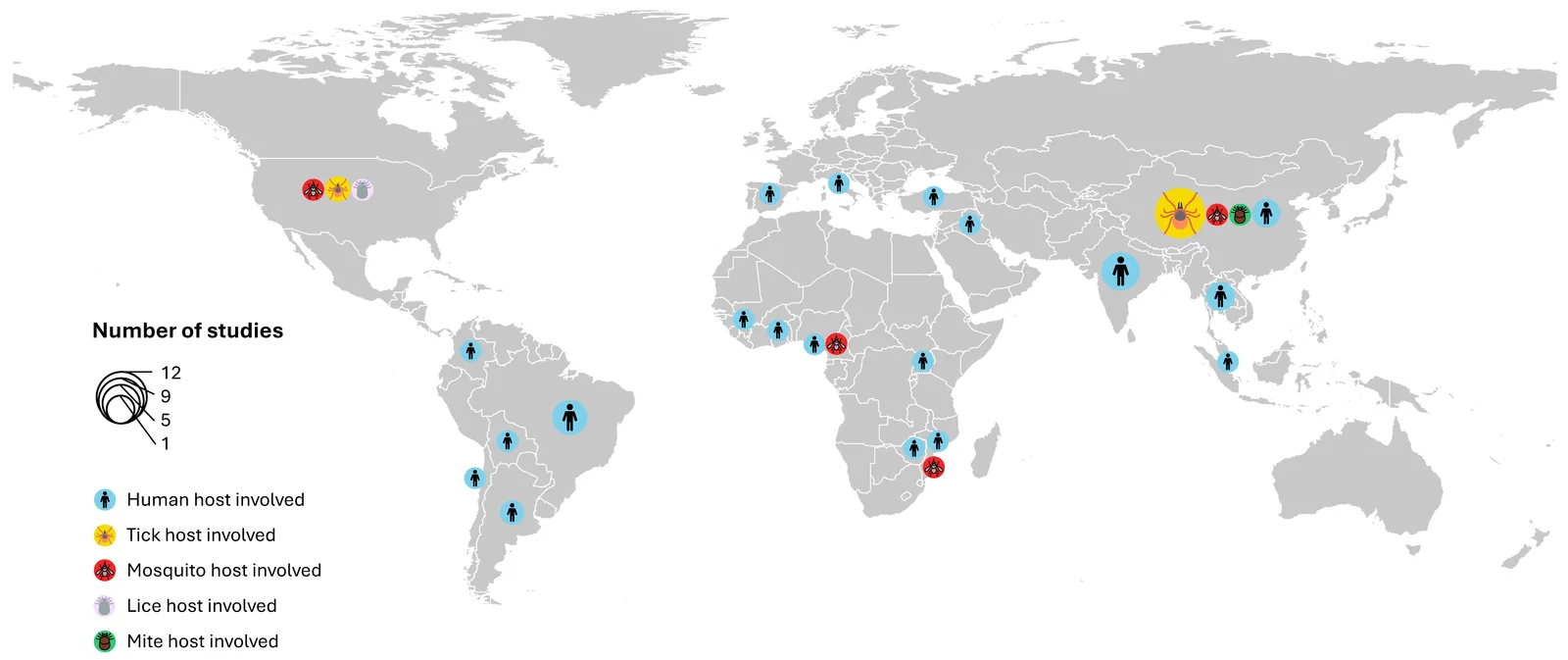
Geographical distribution of the reviewed studies according to the host type.

**Figure 6:**
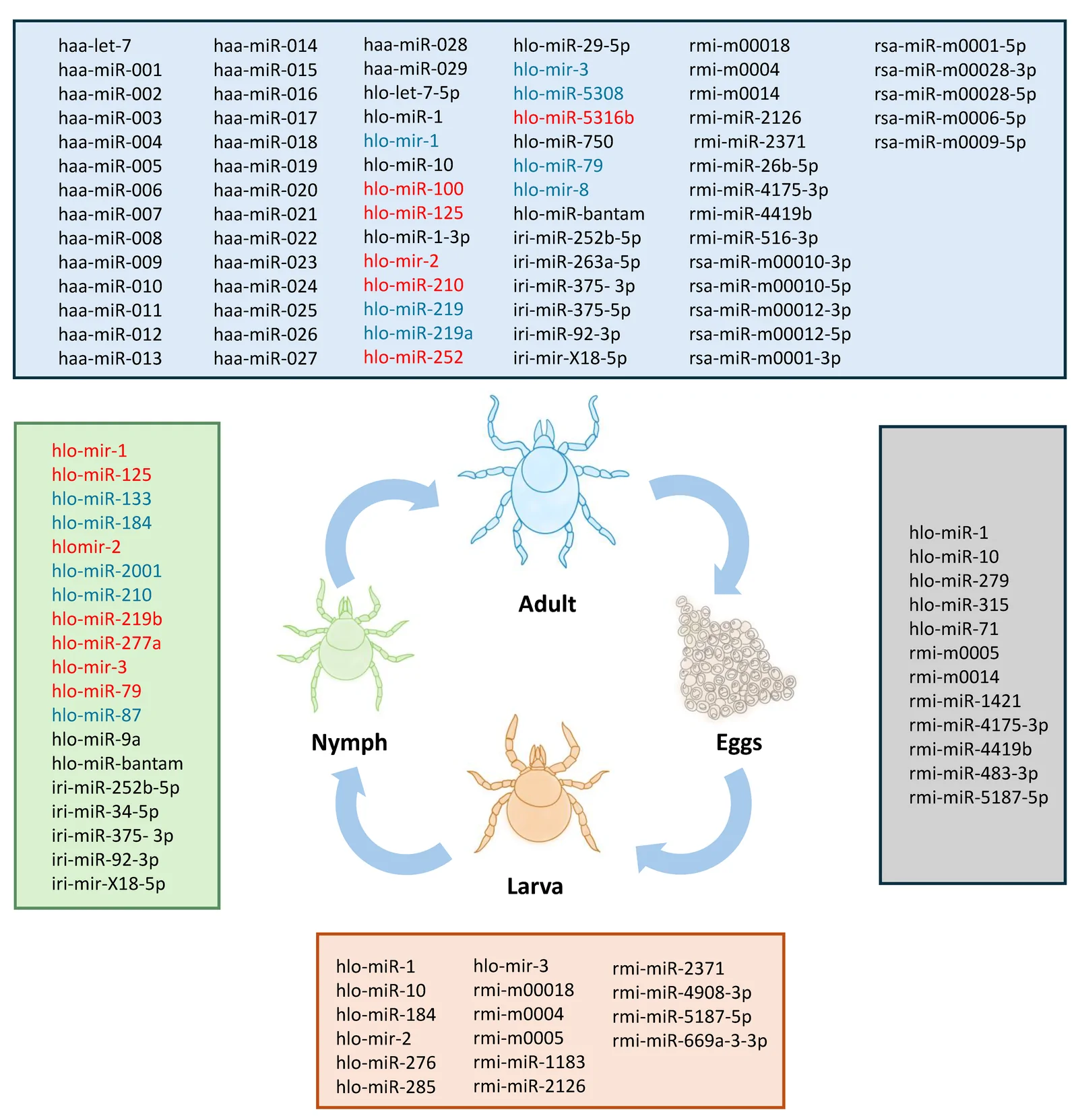
Differentially expressed miRNAs in the tick life cycle from egg to adult. (Statistically significantly upregulated miRNAs are marked in red, and statistically significantly downregulated miRNAs are marked in blue).

**Figure 7:**
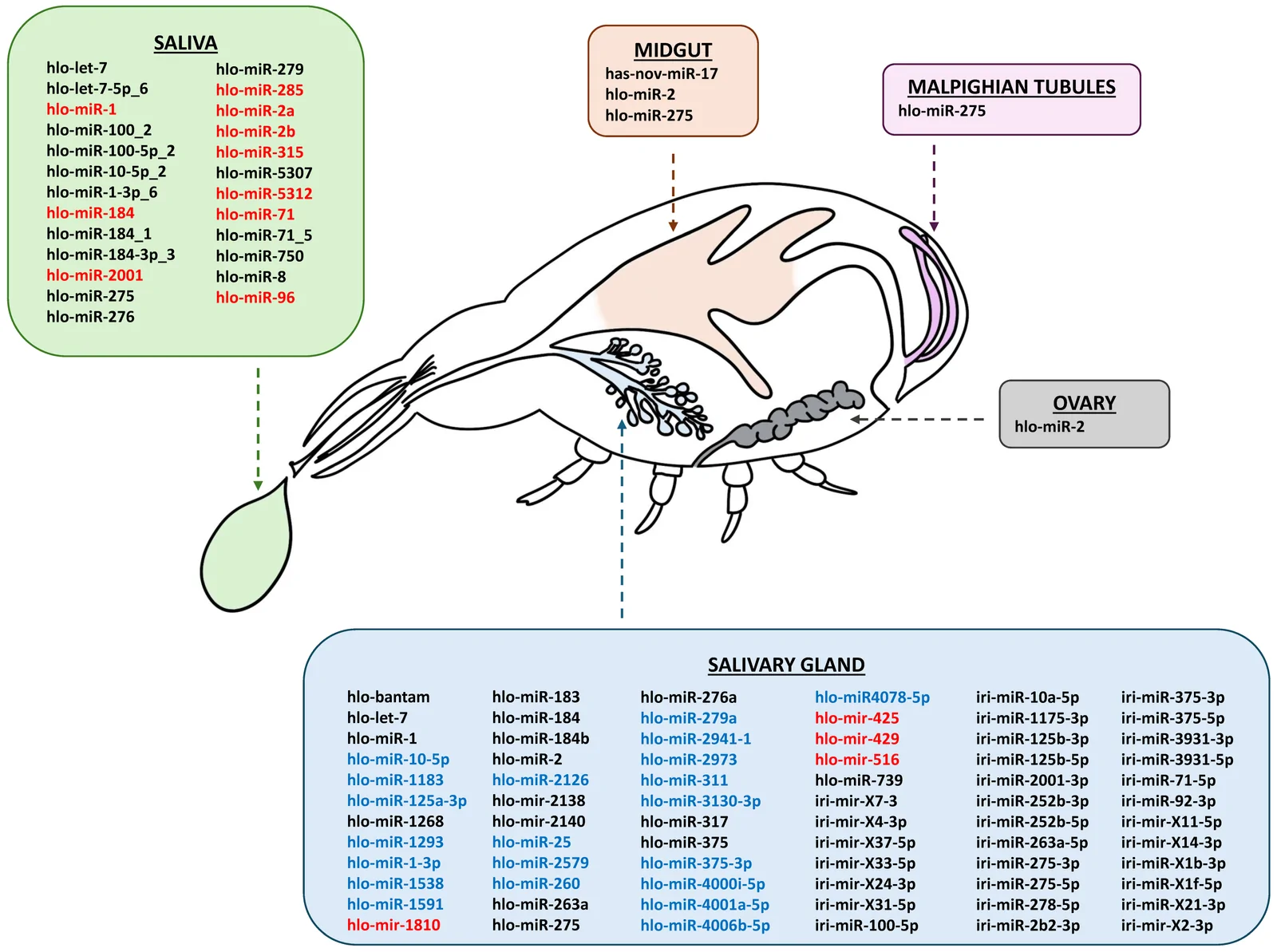
Differentially expressed miRNAs in tick tissues/organs. (Statistically significantly upregulated miRNAs are marked in red, and statistically significantly downregulated miRNAs are marked in blue).

**Table 1:** Characteristics of in vivo human host-based studies to detect differential miRNAs associated with VBDs.

Infectious Agent Type	VBD	Diagnostic Test to Identify VBD	Country (Sample Source)	Cases Sample Characteristics	Gender		Age		Control Sample Characteristics	Sample Type	References
					Male (%)	Female (%)	Paediatrics (%) (<15 Years)	Adults (%) (>15 Years)			
Parasite	African Trypanosomiases	Serology (CATT, trypanolysis, microscopy)	Guinea	32 trypanosomiasis patients (5 CATT+/Para−/PCR−/Lysis+, 7 CATT+/Para−/PCR−/Lysis−, 9 CATT+/Para+/PCR+/Stage I, 11 CATT+/Para+/PCR+/Stage II)	ND	ND	ND	ND	8 serology−, para− healthy individuals	Whole blood	[33]
	Chagas Disease (American trypanosomiasis)	Serology (ELISA), Immunochromatography, Gelatin particle agglutination test	Argentina	38 CD patients (24 CD-non-CCC, 14 CCC)	53%	47%	0%	100%	36 CD− (20 healthy individuals, 16 ischemic cardiomyopathy)	Serum	[34]
		Serology (ELISA, IIA, IHT), ECG, ECHO	Brazil	16 CD patients (10 CCC, 6 DCM)	63%	37%	0%	100%	5 healthy individuals	Human left ventricular free wall heart tissue	[35]
		Serology (IgG), echocardiography, electrocardiography	Colombia	74 CCC patients with reduced left ventricular ejection fraction ≤ 40%	59%	41%	0%	100%	ND	Serum	[36]
		Serology, clinical symptoms (typical heart conduction abnormalities), histopathological diagnosis of myocarditis, fibrosis, and hypertrophy	Brazil	8 CCC patients undergoing heart transplantation	75%	25%	0%	100%	4 non-failing heart tissue samples from healthy individuals	Human left ventricular free wall heart tissue	[37]
		Serology, clinical examination, ECG, ECHO	Brazil	20 CD patients (10 CCC, 10 CDI)	ND	ND	0%	100%	20 healthy individuals	Plasma	[38]
		ECG, Kuschnir score (based on clinical symptoms and ECG)	Chile, Bolivia, and Italy	131 CD patients with abnormal ECGs	32%	68%	ND	ND	81 CD patients with normal ECG findings	Serum	[39]
		Serology (IIA, IHT), symptomatic heart failure, left ventricular ejection fraction ≤ 55%, presence of myocardial fibrosis	Brazil	38 CD patients (28 CD cardiac form with left ventricular dysfunction, 10 CDI)	66%	34%	0%	100%	10 healthy individuals	Plasma/serum exosomes, cardiac tissue	[40]
		Serology (ELISA, hemagglutination, or immunofluorescence), clinical symptoms, chest X-ray, 12-lead resting ECG, and/or echocardiography	Argentina, Spain	40 CD patients (19 indeterminate CD (asymptomatic, with normal ECG and normal chest X-ray), 21 mild and moderate CD cardiomyopathy)	67%	33%	0%	100%	26 *T. cruzi* seronegative healthy individuals	Plasma and serum	[41]
	Malaria	Blood smear testing	ND	19 malaria patients (16 *P. vivax* infected and 3 *P. falciparum* infected)	ND	ND	0%	100%	19 healthy individuals	Plasma	[42]
		Blood smear testing	Mozambique	204 severe malaria patients (*P. falciparum-infected*)	ND	ND	100%	0%	Outpatient children with uncomplicated malaria	Plasma	[43]
		Microscopy, multiplex nested PCR, real-time PCR, LAMP	India	44 malaria patients (22 *P. vivax* (complicated malaria) infected, 20 *P. vivax* (uncomplicated malaria) infected, and 2 *P. falciparum* infected patients)	59%	41%	0%	100%	4 healthy individuals	Whole blood	[44]
		Microscopic examination	Thailand	19 *P. vivax-infected* and 18 *P. falciparum-infected* patients	89%	11%	0%	100%	20 uninfected community individuals	Plasma extracellular vesicles	[45]
	Onchocerciasis	Clinical symptoms (palpable nodules and microfilaridermia confirmed by skin snip)	Cameroon and Ghana	25 infected patients	ND	ND	ND	ND	13 uninfected individuals	Serum and plasma	[46]
	Schistosomiasis	Parasitological detection	China	11 infected patients	ND	ND	ND	ND	6 uninfected individuals	Serum	[47]
		Microscopic examination, faecal/urine test for *Schistosoma* eggs	Zimbabwe and Uganda	25 *Schistosoma* egg-positive individuals	52%	48%	ND	ND	19 *Schistosoma* egg-negative individuals	Serum	[48]
		History of exposure, serology, faecal/urine test for *Schistosoma* eggs	Several countries on the African continent are mentioned under area of exposure	26 schistosomiasis patients	54%	46%	0%	100%	17 healthy individuals who had not travelled to areas of endemicity	Serum and extracellular vesicles	[49]
	Visceral Leishmaniasis (VL)	Parasitological confirmation	Iraq	100 VL-confirmed patients	ND	ND	0%	100%	80 healthy individuals	Whole blood	[50]
Virus	Crimean-Congo haemorrhagic fever (CCHF)	Clinical symptoms, seasonality, evidence of tick bite, contact with infected humans or animals, ELISA and q-PCR	Turkey	25 CCHF patients	ND	ND	ND	ND	6 healthy individuals without CCHF symptoms, and negative serological laboratory test results	Whole blood	[51]
	Dengue	Serology (ELISA: DENV IgG, IgM)	China	12 dengue patients (6 asymptomatic, 6 acute)	50%	50%	0%	100%	6 dengue-negative healthy individuals	Serum exosomes	[52]
		NS1, IgG/IgM screening	Thailand	154 dengue patients (19 in discovery cohort (11 non-severe, 8 severe), 135 in validation cohort (112 non-severe, 23 severe)	46%	54%	ND	ND	44 dengue negative controls (21 healthy controls, 23 Other febrile illness patients)	Serum	[53]
		Clinical symptoms, microscopy	China	43 dengue patients	53%	47%	0%	100%	35 healthy individuals	Serum	[54]
		NS1 antigen detection, serology	India	39 dengue patients	ND	ND	0%	100%	7 other febrile illness controls	Plasma	[55]
		ND	Thailand	60 febrile and convalescent phase confirmed dengue-infected patients	60%	40%	100%	0%	30 paediatric patients diagnosed as acute febrile illness without dengue infection	Serum	[56]
		NS1 antigen detection, PCR, and serology	Singapore	26 dengue-confirmed patients between day 0 and day 14 of/after infection	65%	35%	ND	ND	6 healthy individuals	Whole blood	[57]
	Japanese encephalitis (JE) virus infection	Serology (MAC-ELISA)	India	95 JE-positive patients	70%	30%	44%	56%	95 healthy individuals	Serum	[58]
		Serology (IgM), clinical symptoms of acute encephalitis syndrome	India	31 JE-positive patients	52%	48%	ND	ND	29 healthy individuals negative for JEV IgM	Exosomes from cerebrospinal fluid	[59]
	Zika virus disease	Real-time RT-PCR	Brazil	12 Zika virus-positive patients	25%	75%	0%	100%	6 healthy individuals negative for Zika virus	Plasma	[60]

(Note: CATT: Card agglutination test for trypanosomiasis; CCC: Chronic Chagas disease cardiomyopathy form; CD: Chagas disease; CDI: Chagas disease indeterminate form; DCM: Dilated cardiomyopathy form; ECG: Electrocardiography; ECHO: Echocardiography with Doppler; ELISA: Enzyme-linked immunosorbent assay; IgM: Immunoglobulin M; IHT: Indirect hemagglutination test; IIA: Indirect immunofluorescence assay; Ischemic cardiomyopathy: Negative Chagas test with coronary artery disease, LAMP: Loop-mediated isothermal amplification; Lysis: Trypanolysis test; MAC-ELISA: Immunoglobulin M antibody capture-enzyme-linked immunosorbent assay; ND: No data; Para: Parasite; PCR: Polymerase chain reaction; q-PCR: Quantitative polymerase chain reaction; Stage I: initial hemolymphatic stage of human African trypanosomiasis; Stage II: Late stage of human African trypanosomiasis involving the central nervous system).

**Table 2:** Differentially expressed miRNAs associated with VBDs that have been reported in human-host-based studies. (Statistically significantly upregulated miRNAs are marked in red, and statistically significantly downregulated miRNAs are marked in blue).

Infectious Agent Characteristics			Experimental Model	Sample/Cell Type	Comparison Group/Patient Type	Most Common Differentially Expressed miRNAs (*)	miRNA Profiling Technique	Validated by	References
Type	Species	VBD							
Bacterium	*Borrelia burgdorferi* (strain B31 MI-16)	Lyme Disease	In vitro	Human astrocytes	ND	1 dpi: miR-4792, miR-139-5p 2 dpi: miR-143-3p, miR-135a-3p, miR-135a-5p, miR-146b-5p, miR-212-3p, miR-122-5p, miR-136-5p, miR-199a-5p, miR-4792, miR-145-3p, miR-33b-3p, miR-143-5p, miR-216a-5p, miR-186-3p, miR-133a-3p, miR-18a-3p, miR-1-3p, miR-942-5p, miR-3619-5p, miR-145-5p, miR-185-3p, miR-483-5p, miR-3691-5p, miR-7641, miR-302a-3p, miR-579-5p, miR-656-5p, miR-548ap-3p, miR-3200-5p, miR-4762-5p, miR-376a-2-5p, miR-4797-3p, miR-3622a-5p, miR-545-3p, miR-532-3p, miR-487b-5p, miR-326, miR-3679-5p, miR-146b-1, miR-199a-1, miR-376a-2	RNA sequencing	RT-qPCR	[61]
	*Borrelia burgdorferi* (strain B31 A3)	Lyme Disease	In vitro	Primary dermal fibroblasts	ND	miR-146a-5p	Individual RT-qPCR assays	ND	[62]
	*Coxiella burnetii*	Q fever	In vitro	THP-1 macrophages	ND	miR-708-5p, miR-145-5p, miR-143-3p, miR-106b-5p, miR-181d-5p, miR-16-5p, miR-222-3p, miR-365b-3p, miR-218-5p, miR-125a-5p, miR-192-5p, miR-146a-5p	RNA sequencing	RT-qPCR	[63]
	*Rickettsia parkeri*	Rickettsia	In vitro	Dermal microvascular endothelial cells	ND	miR-23a, miR-27a, miR-24	Stem–loop RT-qPCR	RT-qPCR	[64]
	*Rickettsia australis* (Cutlack strain) (87) and *Rickettsia parkeri* (Atlantic Rainforest strain)	Rickettsia	In vitro	Human umbilical vein vascular endothelial cells (HUVEC)	ND	mir-23a, mir-30b, mir-4488, mir-1468	RNA sequencing	Stem-loop RT-qPCR	[65]
	*Rickettsia rickettsii* (Strain Sheila Smith)	Rickettsia	In vitro	Human dermal microvascular endothelial cells (HMECs)	ND	miR-129-5p, miR-200a-3p, miR-297, miR-200b-3p, miR-595, miR-574-5p, miR-647, miR-615-3p, miR-1224-5p, miR-1238-3p, miR-196a-5p, miR-616-3p, miR-760, miR-1275, miR-146a-5p, miR-631, miR-661, miR-766-3p, miR-1273a, miR-1202, miR-943, miR-1250-5p, miR-1229-3p, miR-301b-3p, miR-548a-3p, miR-377-3p, miR-602, miR-802, miR-376b-3p, miR-216b-5p, miR-216a-5p, miR-410-3p, miR-29b-3p, miR-424, miR-503	Microarray	RT-qPCR	[66]
Parasite	*Babesia divergens* (strain Bd Rouen 1986) and *Babesia microti*	Babesiosis	In vitro	Red blood cells	ND	miR-1290, miR-3p-1228, and miR-3p-8691, miR-183-3p, miR-1277-5p, miR-543	RNA sequencing, microarray	RT-qPCR	[67]
	*Leishmania braziliensis* (BR/18627, BR/30028, and BR/30035)	Leishmaniasis	In vitro	Human monocyte-derived macrophages	Mucosal Leishmaniasis (ML) vs. Cutaneous Leishmaniasis (CL)	miR-103a-3p, miR-21-3p, miR-125a-3p, miR-155-5p, miR-146a-5p, miR-132-5p, miR-147a	RT-qPCR	ND	[68]
					Disseminated Leishmaniasis (DL) vs. CL	miR-155-5p, miR-146a-5p, miR-132-5p, miR-147a			
	*Leishmania donovani* (MHOM/IN/83/AG83)	Leishmaniasis	In vitro	Human THP-1 derived macrophages (TDM) and human Peripheral Blood Mononuclear Cell (PBMC) derived macrophages	ND	miR-15a-5p	RT-qPCR	ND	[69]
	*Leishmania. donovani*	Visceral leishmaniasis	In vivo	Whole blood	Cases vs. Controls	miR-122	Individual RT-qPCR assays	ND	[50]
	*Leishmania major* (Friedlin V1 strain) and *Leishmania donovani* (1S strain)	Leishmaniasis	In vitro	Human monocyte-derived dendritic cells	Dendritic cells infected with *L. major* vs. Uninfected cells	miR-181c, miR-1261, miR-19b, miR-148b, miR-212, miR-301a, miR-551b, miR-193a-5p, miR-99b, miR-193a-3p, miR-101, miR-146b-5p, miR-23b, miR-29c, miR-107, miR-20a, miR-331-3p, let-7d, miR-125b, miR-15a	RNA sequencing	RT-qPCR	[70]
					Dendritic cells infected with *L. donovani* vs. Uninfected cells	miR-338-3p, miR-331-3p, miR-511, miR-193a-3p, miR-744, miR-107, miR-99a, miR-193a-5p, miR-324-3p, miR-941, miR-16, miR-378, miR-18a, miR-28-5p, miR-140-3p, miR-22, miR-15a, miR-423-5p, miR-503, miR-1260			
				Monocyte-derived macrophages	Macrophages infected with *L. major* vs. Uninfected cells	miR-744, miR-193a-3p, miR-181c, miR-24, miR-29b, miR-142-5p, miR-15a, miR-142-3p, miR-1260, miR-103, miR-93, miR-26b, miR-223, miR-193a-5p, let-7a, miR-23b, let-7d, miR-574-5p, miR-1261, miR-16			
					Macrophages infected with *L. donovani* vs. Uninfected cells	let-7b, miR-193a-5p, miR-103, miR-142-3p, miR-223, miR-99b, miR-338-3p, miR-342-3p, miR-24, miR-21, miR-23a, miR-15a, miR-1260, let-7g, miR-26a, miR-107, let-7f, miR-29a, miR-132, miR-16			
	*Leishmania major* (MRHO/IR/75/ER)	Leishmaniasis	In vitro	Monocyte-derived macrophages	Infected macrophages vs. Controls (at 24 hpi and 48 hpi)	let-7a	RT-qPCR	ND	[71]
	*Onchocerca volvulus*	Onchocerciasis	In vivo	Serum and plasma	Cases vs. Controls	miR-71, lin-4, miR-100d, miR-87-3p, miR-100a, bantam	RNA sequencing	ND	[46]
	*Plasmodium falciparum* and *Plasmodium vivax*	Malaria	In vivo	Plasma	Both *P. falciparum* and *P. vivax* cases vs. Controls	miR-451, miR-16, miR-223, miR-296-3p	RT-qPCR	ND	[42]
					*P. falciparum* cases vs. Controls	miR-451, miR-16, miR-223, miR-296-3p			
					*P. vivax* cases vs. Controls	miR-451, miR-16, miR-223, miR-296-3p			
					*P. vivax* cases vs. *P. falciparum* cases	miR-451, miR-16, miR-223, miR-296-3p			
	*Plasmodium falciparum*	Malaria	In vivo	Plasma	Cases vs. Controls	miR-3158–3p, miR-4497	RNA sequencing	RT-qPCR	[43]
					Severe anaemia, prostration and acute respiratory distress (ARD)	miR-122-5p, miR-4492, miR-4497, miR-6087, miR-511-5p			
					Severe anaemia and ARD	miR-4497			
					Severe anaemia vs. Uncomplicated malaria	miR-122–5p, miR-320a, miR-1246, miR-1290, miR-3158–3p			
					Confirmed with prostration, multiple seizures, and acute respiratory distress vs. Uncomplicated malaria	miR-3158–3p, miR-4497			
	*Plasmodium falciparum* and *Plasmodium vivax*	Malaria	In vivo	Whole blood	Cases vs. Controls	miR-7977, miR-28-3p, miR-378a-5p, miR-194-5p, miR-3667-5p, miR-3651, miR-192-5p, miR-19b-3p, miR-181b-5p, miR-29a-3p, miR-200c-3p, miR-155-5p	Microarray analysis	RT-qPCR	[44]
					Complicated *P. vivax* vs. Uncomplicated *P. vivax*	miR-7977, miR-28-3p, miR-378a-5p, miR-194-5p, miR-3667-5p			
	*Plasmodium falciparum* and *Plasmodium vivax*	Malaria	In vivo	Plasma extracellular vesicles	Cases vs. Controls	miR-150-5p, miR-15b-5p, let-7a-5p	RT-qPCR	ND	[45]
	*Schistosoma japonicum*	Schistosomiasis	In vivo	Serum	Cases vs. Controls	miR-223	RT-qPCR	ND	[47]
	*Schistosoma mansoni*	Schistosomiasis	In vivo	Serum	Cases vs. Controls	sma-miR-bantam, sma-miR-277, sma-miR-3479-3p	RT-qPCR	ND	[48]
	*Schistosoma mansoni*, *Schistosoma haematobium*, *Schistosoma mekongi*	Schistosomiasis	In vivo	Serum	Cases vs. Controls	sma-miR-bantam, sma-miR-3488	1-Step Real-Time RT-PCR	ND	[49]
				Extracellular vesicles	Cases vs. Controls	sma-miR-bantam, sma-miR-3488, sma-miR-2c-3p, sma-miR-2a-5p			
	*Trypanosoma brucei gambiense*	African Trypanosomiases	In vivo	Whole blood	Stage II cases vs. Controls	miR-193b, miR-119a-3p, miR-27b, miR-126, miR-98, miR-409-3p, miR-4291, miR-146b-5p, miR-454, miR-195, miR-144, miR-22, miR-374c	Microarray	RT-qPCR	[33]
					Stage I cases vs. Controls	miR-193b, miR-119a-3p, miR-27b, miR-126, miR-98, miR-409-3p, miR-4291, miR-146b-5p, miR-454, miR-338-5p			
	*Trypanosoma cruzi*	Chagas disease (American Trypanosomiasis)	In vivo	Serum	Chronic Chagas disease cardiomyopathy (CCC) vs. Controls	miR-454-5p, miR-130b-3p, miR-651-5p, miR-95-3p, miR-381-3p, miR-1270, miR-324-5p, miR-3940-3p, miR-548au-5p, miR-7-1-3p	RNA sequencing	ND	[34]
	*Trypanosoma cruzi*	Chagas disease	In vivo	Human left ventricular free wall heart tissue	CCC vs. Controls	miR-1, miR-133a-2, miR-133b, miR-208a, miR-208b	RT-qPCR	ND	[35]
					Dilated cardiomyopathy (DCM) vs. Controls	miR-133b, miR-208a, miR-208b			
					CCC vs. DCM	miR-1, miR-133a-2, miR-208b			
	*Trypanosoma cruzi*	Chagas disease	In vivo	Serum	CCC vs. Controls	miR-34a-5p, miR-208a 5p, miR-185-5p, miR-223-5p, let-7d-5p, and miR-454-5p	RT-qPCR	ND	[36]
	*Trypanosoma cruzi*	Chagas disease	In vivo	Human left ventricular free wall heart tissue	CCC vs. Controls	miR-146a-5p, miR-155-5p, miR-151a-3p, miR-22-3p, miR-221-3p, miR-27b-3p, miR-499a-5p, miR-451a, miR-143-3p, miR-494-3p, miR-203a-3p, miR-455-3p	RT-qPCR using TaqMan Low-Density Array microfluidic cards	ND	[37]
	*Trypanosoma cruzi*	Chagas disease	In vivo	Plasma	Chagas disease indeterminate (CDI) form vs. Controls	miR-208a-3p, miR-34a-5p	RT-qPCR	ND	[38]
					CDI vs. CCC	miR-208a-3p, miR-34a-5p			
					CCC vs. controls	miR-208a-3p, miR-34a-5p			
	*Trypanosoma cruzi*	Chagas disease	In vivo	Serum	CD with abnormal ECGs vs. CD with normal ECGs	miR-629-5p, miR-101-3p, miR-576-3p	RNA sequencing	ND	[39]
					CD bundle branch block present vs. absent	miR-101-3p, miR-629-5p, miR-576-3p			
					Higher Kuschnir Score vs. Lower Kuschnir Score	miR-101-3p, miR-576-3p, miR-629-5p			
	*Trypanosoma cruzi*	Chagas disease	In vivo	Plasma/serum exosomes	Serum vs. Plasma samples of CCC	miR-19a-3p, miR-29b-3p, miR-30a-5p	RT-qPCR	RT-qPCR	[40]
					CCC vs. CDI	miR-19a-3p, miR-21-5p, miR-29b-3p			
				Cardiac tissue	CCC vs. Controls	miR-19a-3p, miR-21-5p, miR-29b-3p, miR-30a-5p, miR-199b-5p, miR-208a-3p			
			In vitro	Human cardiac fibroblasts	TGF-β1-stimulated cardiac fibroblasts vs. unstimulated cells	miR-21-5p			
	*Trypanosoma cruzi*	Chagas disease	In vivo	Serum and plasma	Chronic Chagas (ICD and MCC) vs. Controls	miR-224-5p, miR-Contig-1519, miR-Contig-3244, miR-148a-3p	RNA sequencing	RT-qPCR	[41]
Virus	Chikungunya virus (CHIKV Ross Strain E1: A226)	Chikungunya virus disease	In vitro	Primary human synovial fibroblasts	ND	miR-921, miR-4299, miR-4762-5p, miR-1260a, miR-5692c, miR-4454, miR-5692b, miR-21-5p, miR-4796-5p, miR-4775, miR-216a-5p, miR-3074-3p, miR-487b-3p, miR-4797-5p, miR-1260b, miR-4695-3p, miR-491-3p, miR-5581-3p, miR-1264, miR-4717-3p, miR-4286, miR-138-2-3p, miR-5100, miR-4443, miR-382-3p, miR-3182	Microarray analysis	RT-qPCR	[72]
	Chikungunya virus (CHIKV (A226) (MOI 5, African genotype, Strain No. 061573)	Chikungunya virus disease	In vitro	Human skin fibroblast cells (1BR3)	ND	miR-106a, miR-125a, miR-136/136, miR-200a, miR-210, miR-223, miR-370, miR-378, miR-551b, miR-875-3p, miR-99a, miR-16-2, miR-26b, miR-27a, miR-27b, miR-32, and miR-452	RT-qPCR using TaqMan Low-Density Array microfluidic cards	ND	[73]
	Crimean-Congo haemorrhagic fever virus	Crimean-Congo haemorrhagic fever	In vivo	Whole blood	Non-fatal cases vs. Controls	miR-1268a, miR-3940-5p, miR-937-5p, miR-1249-3p, miR-296-5p, miR-144-3p, miR-144-5p, miR- 148a-3p, miR-101-3p, miR-126-3p, miR-96-5p, miR-18a-5p, miR-27a-3p, miR-590-5p, miR-18b-5p	Microarray analysis	RT-qPCR	[51]
					Fatal cases vs. Controls	miR-4793-3p, miR-195-3p, miR-4440, miR-1909-5p, miR-548 am-5p, miR-7850-5p, miR-1908-3p, miR-6775-3p, miR-595, miR-7108-3p, let-7f-5p, let-7a-5p, let-7g-5p, miR-144-3p, miR-142-5p, miR-16-5p, miR-144-5p, miR-20b-5p, let-7d-5p, miR-15b-5p			
					Fatal cases vs. Non-fatal cases	miR-1909-5p, miR-4767, miR-548 am-5p, miR-6511a-3p, miR-4785, miR-6775-3p, miR-7850-5p, miR-6784-3p, miR-4793-3p, miR-4440, miR-7975, miR-142-5p, miR-532-3p, let-7a-5p, miR-425-5p, miR-550a-3-5p, miR-19b-3p, let-7f-5p, miR-223-3p, miR-185-5p, miR-150-5p			
	Dengue virus (DENV2-P23085 INDI-60 strain)	Dengue viral infection	In vitro	Human hepatocellular carcinoma cells (Huh7) and Human embryonic kidney cells (HEK293T)	ND	miR-10b-5p, miR-21-5p, miR-125a-5p, miR-148a-3p, miR-375	RNA sequencing	RT-qPCR	[74]
	Dengue virus	Dengue viral infection	In vivo	Serum exosomes	Acute and asymptomatic dengue vs. Controls	miR-143-3p, miR-1290, miR-125a-5p, miR-10a-5p, miR-10b-5p, miR-182-5p, miR-140-3p, miR-146a-5p, miR-20a-5p, miR-106b-3p, miR-17-5p, miR-425-5p, let-7i-5p, miR-93-5p, miR-378a-3p, miR-151a-3p, miR-152-3p, miR-28-3p, miR-103a-3p, miR-30c-5p, miR-744-5p, miR-122-5p	RNA sequencing	RT-qPCR	[52]
					Asymptomatic dengue vs. Acute dengue	miR-183-5p, miR-363-3p, miR-3615, miR-486-5p, miR-16-2-3p, miR-451a, miR-27a-3p, miR-146b-5p, miR-342-5p, miR-223-3p, miR-142-5p, miR-223-5p, miR-24-3p, miR-32-5p, miR-532-5p, miR-16-5p, miR-101-3p, miR-25-3p			
	Dengue virus (DENV-1,2,3,4, 1 and 3, 1,2, and 3)	Dengue viral infection	In vivo	Serum	Severe Dengue (SD) vs. Dengue Infection (DI)	miR-574-5p, miR-1972, miR-630, miR-890, miR-378g, miR-22-3p, miR-122-5p, miR-193a-5p+miR-193b-5p, miR-30d-5p, miR-320d, miR-30e-5p, miR-30a-5p, miR-665, miR-152-3p, miR-24-3p, miR-593-3p, miR-181a-5p, miR-1305, miR-424-5p, miR-140-5p, let-7i-5p, miR-128-1-5p, miR-610, miR-640, miR-874-3p, miR-3136-5p, miR-502-5p, miR-5196-5p, miR-18a-5p, miR-513a-3p, miR-3161, miR-372-3p, miR-489-3p, miR-604, miR-625-5p, miR-490-3p, miR-196a-3p.	NanoString nCounter Human v3 miRNA Expression Assay	RT-qPCR	[53]
					Dengue with Warning Signs (DWS) vs. Dengue Infection (DI)	miR-424-5p, miR-574-5p, miR-3180, miR-18a-5p, miR-221-3p, miR-335-5p, miR-378g, miR-191-5p, miR-628-3p, miR-548a-3p			
					Severe Dengue (SD) vs. Non-severe Dengue (DI + DWS)	miR-122-5p, miR-574-5p, miR-424-5p, miR-1303, miR-30d-5p, miR-1246, miR-18a-5p, miR-640			
	Dengue virus (DENV3-290 strain)	Dengue viral infection	In vitro	Dendritic cells	ND	miR-1246, miR-320a, miR-3937	RNA sequencing	RT-qPCR	[75]
	Dengue virus (DENV3-5532 strain)					let-7e, miR-1246, miR-1261, miR-320a, miR-371b, miR-3937, miR-4327			
	Dengue virus type 1 (DENV-1)	Dengue viral infection	In vivo	Whole blood	Cases vs. Controls	miR-1972, miR-518a-3p, miR-1538, miR-300, miR-21-5p, miR-589-5p, miR-1537-3p, miR-340-5p, miR-1207-5p, miR-632, miR-934, miR-1227-3p, miR-152-3p, miR-596, miR-149-3p, miR-629-5p, miR-188-5p, miR-23a-5p, miR-590-5p, let-7g-3p, miR-421, miR-200a-3p, miR-375, miR-370-3p, miR-20b-5p, -let-7b-5p, miR-365a-3p	miRNA PCR arrays	RT-qPCR	[54]
	Dengue virus (DENV2 strain)	Dengue viral infection	In vitro	Peripheral blood mononuclear cells	ND	miR-4290, miR-4279, miR-625, let-7e, miR-1290, miR-33a, miR-378, miR-1246, miR-767-5p, miR-320c, miR-720, miR-491-3p, miR-3647, miR-451, miR-4286, miR-106b, miR-20a, miR-30b miR-3653	Microarray analysis	RT-qPCR	[76]
	Dengue virus	Dengue viral infection	In vivo	Plasma	Severe dengue and Dengue with warning signs vs. Uncomplicated dengue patients	miR-486-5p, miR-6795-5p, miR-107, miR-92a-1-5p, miR-3615, miR-103a-3p, miR-103a-2-5p, miR-92a-2-5p, miR-501-5p, miR-320b, miR-378a-5p, miR-22-5p, miR-28-5p, miR-146a-5p, miR-340-5p, let-7i-5p, miR-26b-5p, miR-584-5p, miR-191-5p, miR-126-5p, miR-199b-5p, miR-199a-5p, let-7f-5p, miR-222-5p, miR-26a-5p	RNA sequencing	RT-qPCR	[55]
					Severe dengue vs. Controls, Dengue with warning signs and Uncomplicated dengue patients	miR-6499-5p, miR-122-5p, miR-193b-5p, miR-320c, miR-320a, miR-92b-5p, miR-21-5p, miR-181a-5p, miR-27b-5p, miR-30e-5p, miR-30d-5p, miR-151b, miR-127-5p			
	Dengue virus (DENV-2-IND/P23085/1960 strain)	Dengue viral infection	In vitro	Human hepatocellular carcinoma cells (Huh7), human lung carcinoma cells (A549), human monocytic cells (THP1)	ND	miR-92-a-2-5p, miR-122-5p, miR-107-5p, miR-3615-5p, miR-320a-5p, miR-486-5p, miR-26a-5p and miR-191-5p			
	Dengue virus	Dengue viral infection	In vivo	Blood serum	Cases vs. Controls	miR-126-3p	Individual RT-qPCR assays	ND	[56]
	Dengue virus	Dengue viral infection	In vivo	Whole blood	Cases vs. Controls	miR-299-3p	Microarray analysis	RT-qPCR	[57]
					All dengue patients	miR-19a-3p, miR-19b-3p, miR-33b-5p, miR-145-3p, miR-299-3p, miR-320a, miR-374a-5p, miR-423-3p, miR-548e, miR-576-5p, miR-584-5p, miR-614, miR-634, miR-652-3p miR-4451, miR-4503, miR-4529-3p, miR-4788, miR-5583-5p			
					Dengue Haemorrhagic Fever (DHF) vs. Controls	miR-126-3p			
					Acute dengue (0-4 days) vs. Controls	miR-1204, miR-1225-5p, miR-3121-3p, miR-4259, miR-4327, miR-450b-5p, miR-491-5p, miR-499a-3p, miR-512-5p, miR-615-5p, miR-624-5p, miR-892b			
	Japanese encephalitis (JE) virus infection	Japanese encephalitis viral infection	In vivo	Blood serum	Cases vs. Controls	miR-21, miR-155	Individual RT-qPCR assays	ND	[58]
					Non-JE acute encephalitis syndrome vs. JE	miR-21, miR-155			
					Non-JE acute encephalitis syndrome vs. Controls	miR-21, miR-155			
	JE virus infection	Japanese encephalitis viral infection	In vivo	Exosomes from cerebrospinal fluid (CSF)	Cases vs. Controls	miR-150-5p, miR-342-3p, miR-21-5p, miR-32-5p, miR-101-3p, let-7 g-5p, miR-146a-5p, miR-181a-5p, miR-29c-3p, miR-320a, miR-451a, miR-652-3p, miR-99a-5p, miR-182-5p, miR-204-5p, miR-125b-5p	CSF exosomes focused miRNA PCR panels	RT-qPCR	[59]
	JE virus (P20778 strain)	Japanese encephalitis viral infection	In vitro	Human microglial cells (CHME3)	ND	mir-320e, mir-3648, mir-3687, mir-4508, mir-4722, mir-4734, mir-4763, mir-572, mir-129-5p, mir-1913, mir-4526, mir-197-3p	Microarray analysis (Affymetrix miRNA 3.0)	RT-qPCR	[77]
	Severe fever with thrombocytopenia syndrome virus	Severe fever with thrombocytopenia syndrome	In vitro	Human monocyte cell line (THP-1)	ND	miR-146a, miR-146b	RNA sequencing	ND	[78]
	Tick-borne encephalitis virus (TBEV strain Neudorf and strain Hypr)	Tick-borne encephalitis	In vitro	Human neural stem cells	ND	miR-592	RNA sequencing	RT-qPCR	[79]
	West Nile virus (Kunjin strain)	West Nile virus	In vitro	Human embryonic kidney cells (HEK293)	ND	miR-25-3p, miR-151b, miR-24-3p, miR-1307-3p, miR-532-5p, miR-1271-5p, miR-22-3p	RNA sequencing	RT-qPCR, Northern blotting	[80]
	Zika virus	Zika virus disease	In vivo	Plasma	Zika virus-infected cases vs. Controls	miR-365-3p, miR-340-5p, miR-342-3p, miR-30c-5p, let-7a-5p, let-7e-5p, miR-146b-5p, miR-126a-3p, miR-139-5p, miR-19b-3p, miR-24-3p, miR-320b, miR-146a-5p, miR-186-5p, miR-142-3p, miR-30b, miR-199a-3p, miR-125b-5p, miR-191-5p, miR-223-3p, miR-344a-5p, miR-28-3p, miR-150-5p, miR-130a-3p	RT-qPCR	RT-qPCR	[60]
					Zika virus-infected recovery cases (2-3 weeks) vs. Controls	miR-495-3p, miR-17-5p, miR-146a-5p, miR-342-3p, miR-126a-3p, miR-150-5p, miR-30c-5p, miR-30b			
	Zika virus (Brazilian strain PE (ZIKV PE/243 strain)	Zika virus disease	In vitro	SH-SY5Y neuroblastoma cell line (ATCC CRL266)	ND	miR-145,miR-126, miR-1267, miR-99, miR-190b, miR-299-5p, miR-361-3p, miR-522, miR-148a, miR-342-5p, miR-598, miR-708-3p	RT-qPCR	RT-qPCR	[81]
	Zika virus (ZIKV strain Uganda 976 and strain PF13/251013-18)	Zika virus disease	In vitro	Long-term self-renewing neuroepithelial-like stem cells	ND	miR-6786-5p, miR-6075, miR-6090, miR-4667-5p, miR-6084, miR-182-5p	RNA sequencing	RT-qPCR	[82]

(*) Displayed are the top differentially expressed miRNAs. The full dataset is available in Supplementary Table S4. (Note: ARD: Acute respiratory distress; CCC: Chronic Chagas disease cardiomyopathy form; CD: Chagas disease; CDI: Chagas disease indeterminate form; DCM: Dilated cardiomyopathy form; ND: No data; PCR: Polymerase chain reaction; q-PCR: Quantitative polymerase chain reaction; RT-qPCR: Reverse transcription quantitative real-time polymerase chain reaction; RNA: Ribonucleic acid; Stage I: initial hemolymphatic stage of human African trypanosomiasis; Stage II: Late stage of human African trypanosomiasis involving the central nervous system).

**Table 3:** Differentially expressed miRNAs in different vector species associated with VBDs. (Statistically significantly upregulated miRNAs are marked in red and statistically significantly downregulated miRNAs are marked in blue).

Vector Type	Vector Characteristics			Infectious Agent (IA) Characteristics			Most Common Differentially Expressed miRNAs	miRNA Profiling Technique	Validated by	Reference
	Species	Vector Sample Type	Location	IA Type	Species	VBD				
Mosquito	*Aedes aegypti*	Uninfected saliva	Tahiti, French Polynesia	Virus	Chikungunya virus	Chikungunya virus disease	miR-33-5p, miR-285-3p, miR-21766	RNA sequencing	ND	[87]
		Infected saliva	Tahiti, French Polynesia	Virus	Chikungunya virus	Chikungunya virus disease	miR-1175-5p, miR-2943-1-5p			
	*Aedes aegypti*	Adult female midgut	China	Virus	Zika virus (Jiegao strain)	Zika Virus disease	2dpi: miR-2946, miR-989, miR-2941, miR-309a, miR-375 4dpi: miR-2946, miR-989, miR-2941, miR-309a, miR-375 6dpi: miR-2946, miR-989, miR-2941	RNA sequencing	RT-qPCR	[88]
			China	Virus	Zika virus (Mengding strain)	Zika Virus disease	2dpi: miR-286b 4dpi: miR-2946, miR-2941 6dpi: miR-2946, miR-989			
		Adult female salivary gland	China	Virus	Zika virus (Jiegao strain)	Zika Virus disease	2dpi: miR-309a, miR-286b, miR-279, miR-2941 4dpi: miR-375, miR-932-5p, miR-957, miR-210, miR-285, miR-932-3p, miR-307 6dpi: miR-375			
			China	Virus	Zika virus (Mengding strain)	Zika Virus disease	2dpi: miR-2946, miR-989, miR-2941, miR-309a, miR-375, miR-286b, miR-279, miR-932-5p, miR-957, miR-210, miR-285 4dpi: miR-375 6dpi: miR-285, miR-2946, miR-294			
	*Aedes aegypti*	Adult female mosquitoes	China	Virus	Zika virus	Zika Virus disease	2dpi: miR-308-5p, miR-989, miR-263a-5p, miR-286a, miR-2941, miR-2944b-3p, miR-2944b-5p, miR-2946, miR-309a, miR-980-3p 7dpi: miR-2940-3p, mir-1-5p, miR-286b, miR-2941, miR-308-3p, miR-308-5p, miR-375 14dpi: miR-71-5p, miR-989, miR-286b, miR-305-5p, miR-308-3p, miR-309a	RNA sequencing	RT-qPCR	[89]
	*Aedes aegypti*	*Aedes aegypti cell line (Aag2)*	N/A	Parasite	*Brugia malayi*	Filariasis	aae-mir-1175, aae-mir-2945, bmo-mir-6497, nlo-mir-275, aae-mir-184, and PC-5p-30141_33	RNA sequencing	RT-qPCR	[90]
	*Aedes aegypti*	Adult mosquito midgut	Laboratory colony	Virus	Ross River virus (T48 strain)	Ross River virus disease	2 dpi: miR-9b-5p	RNA sequencing	RT-qPCR	[91]
		Adult mosquito fat body tissues					2 dpi: miR-9b-5p, miR-2945-3p, miR-9a-5p, miR-989-3p, miR-317-3p, miR-275-3p, miR-275-5p, miR-8-5p, miR-988-3p, miR-34-3p, miR-184-3p, miR-2940-5p 12 dpi: miR-2940-3p, miR-71-3p			
	*Aedes aegypti, Aedes albopictus*	Adult female mosquitoes	ND	Virus	Dengue virus (DENV-2) and Zika virus	Dengue and Zika virus diseases	miR-23a-3p, miR-27a-3p, miR-128-3p, miR-143-3p, miR146a-3p, miR150-3p, miR378a-3p, miR-423-3p	RNA sequencing	RT-qPCR	[92]
	*Aedes albopictus*	Aedes albopictus Cell line (C6/36-HT cells and derivatives)	NA	Virus	Dengue virus (DENV-2)	Dengue virus disease	miR-927	RT-qPCR	Dual-luciferase reporter assay; functional gain/loss-of-function assays; RT-qPCR of target and effector genes	[93]
	*Aedes albopictus*	Aedes albopictus Cell line (C6/36)	NA	Virus	Dengue virus (DENV-2)	Dengue virus disease	aal-miR-306, aal-miR-308-1, aal-miR-308-2, aal-miR-12, aal-miR-7-1, aal-miR-7-2, aal-miR-278-1, aal-miR-278-2, aal-miR-263a-2, aal-miR-9c, aal-miR-2a-1, aal-miR-2a-2, aal-miR-2a-3, aal-miR-263a-1, aal-miR-new19-2, aal-miR-new19-1, aal-miR-new6, aal-miR-9b, aal-miR-1, aal-miR-275, aal-miR-new25-1, aal-miR-190, aal-miR-71-2, aal-miR-71-3, aal-miR-71-1, aal-miR-317, aal-miR-13-2, aal-miR-13-1, aal-miR-13-3, aal-miR-279, aal-miR-new7-2, aal-miR-new7-1, aal-miR-277, aal-miR-988, aal-miR-996, aal-miR-219, aal-miR-new15-2, aal-miR-new15-4, aal-miR-new15-1, aal-miR-new15-3, aal-miR-31-3, aal-miR-31-2, aal-miR-31-1, aal-miR-125-2, aal-miR-125-1, aal-miR-980a, aal-miR-980b, aal-miR-new1-3, aal-miR-new1-2, aal-miR-new1-1, aal-miR-989-2, aal-miR-989-1, aal-miR-375-1, aal-miR-2945-1, aal-miR-2945-2, aal-miR-375-3, aal-miR-375-2, aal-let-7-2, aal-let-7-1, aal-miR-2b-1, aal-miR-276b-3, aal-miR-2b-2, aal-miR-276b-4, aal-miR-2b-3, aal-miR-276b-2, aal-miR-2a-6, aal-miR-2a-5, aal-miR-276b-1, aal-miR-2a-4, aal-miR-998-2, aal-miR-998-1, aal-miR-276a-2, aal-miR-276a-1, aal-miR-5-3, aal-miR-5-2, aal-miR-new13-2, aal-miR-5-1, aal-miR-new13-1, aal-miR-981a, aal-miR-281, aal-miR-34, aal-miR-981b, aal-miR-10, aal-miR-184-1, aal-miR-184-2, aal-miR-92b-2, aal-miR-92b-1, aal-miR-137-1, aal-miR-137-2, aal-miR-927-1, aal-miR-927-2, aal-miR-932, aal-miR-970, aal-miR-11-1, aal-miR-11-2, aal-miR-285, aal-bantam	RNA sequencing	ND	[94]
	*Aedes albopictus*	Adult female mosquito midgut	China	Virus	Dengue virus (DENV-2, New Guinea C strain)	Dengue virus disease	5 dpi: let-7f, miR-1260, miR-34-3p, miR-622, miR-2-5p, miR-4448, miR-6666-3p 7 dpi: let-7f, miR-1260, miR-1420b-5p, miR-1767, miR-193-5p, miR-1951, miR-19c, miR-276-3p, miR-2941, miR-2944b-5p, miR-2946, miR-3811e-5p, miR-4110-5p, miR-424-3p, miR-4728-5p, miR-6134, miR-622, miR-976-5p, miR-989, miR-10-3p, miR-1273f, miR-1957b, miR-241-5p, miR-2951-5p, miR-3809-5p, miR-4175-3p, miR-4448, miR-6666-3p 10 dpi: miR-1, miR-100, miR-11, miR-12, miR-1260, miR-14, miR-1420b-5p, miR-15-3p, miR-1767, miR-19c, miR-276-3p, miR-2a-3p, miR-2c, miR-317, miR-34-3p, miR-3870-5p, miR-4110-5p, miR-424-3p, miR-4728-5p, miR-5706, miR-6134, miR-996, miR-998-5p, miR-33	RNA sequencing	Stem-loop RT-qPCR	[95]
	*Anopheles coluzzi*	Adult female mosquito	Cameroon	Virus	*Alphavirus* (O’nyong-nyong virus)	O’nyong-nyong virus disease	aga-chr2L_23370	RNA sequencing	ND	[96]
	*Anopheles gambiae*	Adult female mosquito midgut	ND	Protozoa	*Plasmodium falciparum*, *Plasmodium berghei*	Malaria	aga-miR-8-3p, aga-miR-34-5p, aga-miR-989-3p, aga-miR-14-3p, aga-miR-277-3p, and aga-miR-305-5p	RNA sequencing	ND	[97]
	*Anopheles stephensi*	Adult female mosquitoes	ND	Virus	*Alphavirus* Mayaro virus	Mayaro virus disease	as-mirNOV10, as-mirNOV16, as-mirNOV17, aga-miR-286b, agamiR-2944a, aga-miR-2944b, aga-miR-307, and aga-miR-309.	RNA sequencing	ND	[98]
Tick	*Ixodes scapularis*	Salivary gland	Texas	Flavivirus	Powassan virus (POWV)	Powassan virus disease	mir-X1b, miR-1, miR-315, miR-5307, miR-79, miR-210, miR-12, miR-317, miR-5312	RNA sequencing	RT-qPCR	[99]
	*Ixodes scapularis*	Salivary gland and midgut	Oklahoma	Bacterium	*Borrelia burgdorferi*	Lyme disease	miR-87, miR-71, miR-5307, miR-2001, miR-5310, miR-317, nDS_002784743.1_12165, nDS_002664372.1_28027, nDS_002716687.1_21278, nDS_002620414.1_33611, miR-5310, nDS_002680650.1_26098, miR-79, miR-1, miR-153, miR-9a-5p, miR-1-3p, nDS_002871802.1_376, nDS_002633080.1_31953, nDS_002549652.1_43448, nDS_002537755.1_45711, nDS_002537755.1_45739, nDS_002861213.1_1750, nDS_002763926.1_14582, nDS_002548557.1_43749	RNA sequencing	RT-qPCR	[100]
	*Ixodes scapularis*	Larvae and nymphs	USA	Bacterium	*Anaplasma phagocytophilum*	Anaplasmosis	miR-133	RT-qPCR	ND	[101]
	*Ixodes scapularis*	*Ixodes scapularis* embryo-derived tick ISE6 cell	NA	Bacterium	*Anaplasma phagocytophilum*	Granulocytic anaplasmosis	mir-79, mir-185, mir-7544, mir-9771b, mir-2951, mir-B8, mir-491	RNA sequencing	RT-qPCR	[102]
Tsetse fly	*Glossina morsitans morsitans*	Cardia and midgut tissues	Laboratory colony (Yale University insectary)	Parasite	*Trypanosoma brucei rhodesiense*	African trypanosomiasis	gmo-miR-275-3p, gmo-miR-184-3p, gmo-miR-263b-5p, 6962, gmo-miR-1000-5p	RNA sequencing	RT-qPCR	[103]
	*Glossina morsitans morsitans*	Salivary glands and saliva	Laboratory colony (Institute of Tropical Medicine, Antwerp, Belgium)	Parasite	*Trypanosoma brucei brucei*	African trypanosomiasis	let-7, miR-8, miR-184, miR-29, miR-31, miR-279, miR-34, miR-277, miR-216, miR-276, miR-1, miR-375, miR-7, miR-33, miR-190, miR-252, miR-927, miR-315, miR-87, miR-970, miR-10, miR-305, miR-316, miR-2 (45766>45837), miR-9, miR-bantam, miR-2	RNA sequencing	De novo miRNA prediction	[104]

(Note: NA: Not applicable; ND: No data).

## Data Availability

No new data were created or analyzed in this study. Data sharing is not applicable to this article.

## Supplementary Materials

The following supporting information can be downloaded at: https://www.mdpi.com/article/10.3390/ijms27198444/s1. References [145,146,147,148,149,150,151,152,153,154,155,156,157,158,159,160,161,162,163,164,165,166,167] are cited in Supplementary Materials.

## Abbreviations

The following abbreviations are used in this manuscript:

ARD	Acute Respiratory Distress
AUC	Area Under the Receiver Operating Characteristic Curve
CATT	Card Agglutination Test for Trypanosomiasis
CCC	Chronic Chagas disease Cardiomyopathy form
CCHF	Crimean-Congo Haemorrhagic Fever
CD	Chagas Disease
CDI	Chagas Disease Indeterminate form
CHIKV	Chikungunya Virus
CI	Confidence Interval
CL	Cutaneous Leishmaniasis
CSF	Cerebrospinal Fluid
DCM	Dilated Cardiomyopathy form
DENV	Dengue Virus
DL	Disseminated Leishmaniasis
ECG	Electrocardiography
ECHO	Echocardiography with Doppler
ELISA	Enzyme-linked Immunosorbent Assay
HMECs	Human dermal Microvascular Endothelial Cells
HUVEC	Human Umbilical Vein Endothelial Cells
IgM	Immunoglobulin M
IHT	Indirect Hemagglutination Test
IIA	Indirect Immunofluorescence Assay
JE	Japanese Encephalitis
LAMP	Loop-mediated isothermal Amplification
MAC-ELISA	Immunoglobulin M Antibody Capture Enzyme-linked Immunosorbent Assay
miRNAs	microRNAs
ML	Mucosal Leishmaniasis
NA	Not Applicable
ND	No Data
ONNV	O’nyong-nyong Virus
PAMPs	Pathogen-Associated Molecular Patterns
Para	Parasite
PBMC	Human Peripheral Blood Mononuclear Cell
PCR	Polymerase Chain Reaction
POWV	Powassan Virus
PRISMA	Preferred Reporting Items for Systematic Reviews and Meta-Analyses
q-PCR	Quantitative Polymerase Chain Reaction
RT-PCR	Reverse Transcription Polymerase Chain Reaction
SFTS	Severe Fever with Thrombocytopenia Syndrome
TBEV	Tick-Borne Encephalitis Virus
TDM	Human THP-1-Derived Macrophages
VBDs	Vector-Borne Diseases
VL	Visceral Leishmaniasis
