# Supplementary material for: MicroRNA Responses Associated with Vector-Borne Diseases in Humans and Arthropod Vectors: A Systematic Review
Source: Int J Mol Sci. 2026 Sep 22;27(19):8444. doi: 10.3390/ijms27198444 (PMC13664826; doi:10.3390/ijms27198444)
Supplement: Supplementary file 1 [file ijms-27-08444-s001.zip › Supplementary Table S3. Comprehensives search strategy.pdf]

Supplementary Table S3. Comprehensives search strategy used for identifying articles.

Database	Search strategy with Boolean operators	Filters used	Date accessed	Number of results
**Database search (Phase 1)**				
Web of Science	ALL=("micro RNA" OR "miRNA" OR "microRNA*") AND ALL=("ectoparasite" OR "vector borne" OR "vector-borne" OR "tick" OR ticks OR "mite" OR mites OR "lice" OR "louse" OR "flea" OR fleas OR flea borne OR fleaborne)	Included: Language: English; Publication year: 2003-2025; Document type: Articles.<br>Excluded: Document type: Review Article, Meeting Abstract, Book Chapters, Letter, Editorial Material, Early Access; Language: Indonesian, Spanish	22.12.2025	281
CAB Abstracts	(("Micro RNA" OR "miRNA" OR "microRNA*")) AND (("ectoparasite borne" OR "ectoparasite-borne" OR "vector borne" OR "vector-borne" OR tick OR ticks OR mite OR mites OR lice OR louse OR flea OR fleas OR fleaborne OR "flea borne"))	No filters used	22.12.2025	178
Scopus	(TITLE-ABS-KEY (("micro rna" OR "mirna" OR "microrna*")) AND TITLE-ABS-KEY (("ectoparasite borne" OR "ectoparasite-borne" OR "vector borne" OR "vector-borne" OR tick OR ticks OR mite OR mites OR lice OR louse OR flea OR fleas OR fleaborne OR "flea borne")))	Included: Language: English; Publication year: 2003-2025; Document type: Articles<br>Excluded: Note, Letter, Short survey, Editorial, Book chapter, Review); Languages excluded: Chinese, Croatian, Spanish<br>Filter string: (EXCLUDE (DOCTYPE,"re") OR EXCLUDE (DOCTYPE,"ch") OR EXCLUDE (DOCTYPE,"ed") OR EXCLUDE (DOCTYPE,"sh") OR EXCLUDE (DOCTYPE,"le") OR EXCLUDE (DOCTYPE,"no")) AND (EXCLUDE (LANGUAGE,"Chinese") OR EXCLUDE (LANGUAGE,"Croatian") OR EXCLUDE (LANGUAGE,"Spanish"))	22.12.2025	250
PubMed	(("Micro RNA" OR "miRNA" OR "microRNA*")) AND (("ectoparasite borne" OR "ectoparasite-borne" OR "vector borne" OR "vector-borne" OR tick OR ticks OR mite OR mites OR lice OR louse	Filters: English, Exclude preprints	22.12.2025	226

	OR flea OR fleas OR fleaborne OR "flea borne"))<br>Filters: English			
**Database search (Phase 2)**				
Web of Science	ALL=("micro RNA" OR "miRNA" OR "microRNA*") AND ALL=("mosquito*" OR "sand fly" OR "sandfly" OR "tsetse fly" OR "triatomine" OR "triatomine bug" OR "blackfly" OR "black fly")	Included: Language: English; Publication year: 2003-2025; Document type: Articles. Excluded: Document type: Review Article, Meeting Abstract, Book Chapters, Letter, Editorial Material, Early Access; Language: Indonesian, Spanish	02.07.2026	293
CAB Abstracts	(("Micro RNA" OR "miRNA" OR "microRNA*")) AND (("mosquito*" OR "sand fly" OR "sandfly" OR "tsetse fly" OR "triatomine" OR "triatomine bug" OR "blackfly" OR "black fly"))	No filters used	02.07.2026	224
Scopus	(TITLE-ABS-KEY(("micro rna" OR "mirna" OR "microrna*")) AND TITLE-ABS-KEY(("mosquito*" OR "sand fly" OR "sandfly" OR "tsetse fly" OR "triatomine" OR "triatomine bug" OR "blackfly" OR "black fly")))	Included: Language: English; Publication year: 2003-2025; Document type: Articles<br>Excluded: Note, Letter, Short survey, Editorial, Book chapter, Review); Languages excluded: Chinese, Croatian, Spanish<br>Filter string: (EXCLUDE (DOCTYPE,"re") OR EXCLUDE (DOCTYPE,"ch") OR EXCLUDE (DOCTYPE,"ed") OR EXCLUDE (DOCTYPE,"sh") OR EXCLUDE (DOCTYPE,"le") OR EXCLUDE (DOCTYPE,"no")) AND (EXCLUDE (LANGUAGE,"Chinese") OR EXCLUDE (LANGUAGE,"Croatian") OR EXCLUDE (LANGUAGE,"Spanish"))	02.07.2026	301
PubMed	(("Micro RNA" OR "miRNA" OR "microRNA*")) AND (("mosquito*" OR "sand fly" OR "sandfly" OR "tsetse fly" OR "triatomine" OR "triatomine bug" OR "blackfly" OR "black fly"))	Filters: English, Exclude preprints	02.07.2026	287

Note: Following peer review, the search strategy was expanded to include additional vector-borne disease vectors (mosquitoes, sand flies, tsetse flies, triatomine bugs, and blackflies) during the database search (phase 2) to ensure exhaustive coverage of the literature. All searches employed consistent Boolean operators and methodology adapted to each database's syntax requirements.
