# Supplementary material for: MicroRNA Responses Associated with Vector-Borne Diseases in Humans and Arthropod Vectors: A Systematic Review
Source: Int J Mol Sci. 2026 Sep 22;27(19):8444. doi: 10.3390/ijms27198444 (PMC13664826; doi:10.3390/ijms27198444)
Supplement: Supplementary file 1 [file ijms-27-08444-s001.zip › Supplementary Table S4. Full dataset human studies.pdf]

Supplementary Table S4. Full dataset of differentially expressed miRNAs associated with VBDs that have been reported in human-host-based studies.

Infectious agent characteristics			Experimental model	Sample/ cell type	Comparison group/patient type	Differentially expressed miRNAs	miRNA profiling technique	Validated by	References
**Type**	**Species**	**VBD**							
**Parasite**	*Leishmania major* (Friedlin V1 strain) and *Leishmania donovani* (1S strain)	Leishmaniasis	*In vitro*	Human monocyte-derived dendritic cells	Dendritic cells infected with *L. major* vs. Uninfected cells	miR-19b, miR-148b, miR-181c, miR-193a-5p, miR-212, miR-301a, miR-551b, miR-1261, let-7a, let-7b, let-7c, let-7d, let-7f, let-7g, let-7i, miR-15a, miR-15b, miR-16, miR-18a, miR-19a, miR-20a, miR-21, miR-22, miR-23a, miR-23b, miR-24, miR-25, miR-26a, miR-26b, miR-28-3p, miR-29a, miR-29b, miR-29c, miR-30b, miR-30c, miR-30e, miR-92a, miR-93, miR-99a, miR-101, miR-103, miR-106a, miR-106b, miR-107, miR-125a-5p, miR-125b, miR-128, miR-130b, miR-132, miR-140-3p, miR-142-3p, miR-142-5p, miR-146a, miR-146b-3p, miR-146b-5p, miR-150, miR-151-5p, miR-155, miR-181a, miR-185, miR-191, miR-197, miR-210, miR-221, miR-222, miR-223, miR-326, miR-328, miR-331-3p, miR-338-3p, miR-339-3p, miR-339-5p, miR-342-3p, miR-342-5p, miR-361-3p, miR-362-3p, miR-374a, miR-378, miR-422a, miR-423-3p, miR-423-5p, miR-424, miR-425, miR-484, miR-502-3p, miR-503, miR-532-3p, miR-532-5p, miR-574-5p, miR-652, miR-660, miR-744, miR-1246, miR-1260, miR-1285, miR-1307	RNA sequencing	RT-qPCR	[1]
					Dendritic cells infected with *L. donovani* vs. Uninfected cells	let-7a, let-7b, let-7f, let-7i, miR-25, miR-92a, miR-99a, miR-103, miR-107, miR-185, miR-193a-3p, miR-193a-5p, miR-197, miR-221, miR-324-3p, miR-331-3p, miR-338-3p, miR-342-3p, miR-424, miR-511, miR-744, miR-941, miR-15a, miR-16, miR-18a, miR-22, miR-23a, miR-28-5p, miR-140-3p, miR-191, miR-378, miR-423-5p, miR-503, miR-1260			
				Monocyte-derived macrophages	Macrophages infected with *L. major* vs. Uninfected cells	miR-15a, miR-24, miR-29b, miR-142-3p, miR-142-5p, miR-181c, miR-193a-3p, miR-744, miR-1260, let-7a, let-7d, miR-16, miR-22, miR-23b, miR-25, miR-26a, miR-26b, miR-93, miR-125a-5p, miR-191, miR-193a-5p, miR-197, miR-210, miR-223, miR-331-3p, miR-423-3p, miR-574-5p, miR-1261			
					Macrophages infected with *L. donovani* vs. Uninfected cells	let-7b, miR-21, miR-24, miR-99b, miR-103, miR-142-3p, miR-193a-5p, miR-223, miR-338-3p, miR-342-3p, let-7f, let-7g, miR-15a, miR-16, miR-23a, miR-23b, miR-26a, miR-29a, miR-29b, miR-107, miR-125a-5p, miR-132, miR-148b, miR-191, miR-423-3p, miR-574-5p, miR-1260			
	Trypanosoma cruzi	Chagas disease (American Trypanosomiasis)	*In vivo*	Serum	Chagas non-cardiomyopathy controls vs. Healthy controls	miR-548q, miR-125b-2-3p, miR-23a-5p, miR-511-5p, miR-4662a-5p, miR-3138, miR-6511b-3p, miR-532-3p, miR-1306-5p, miR-7-5p, miR-224-5p, miR-1976, miR-421, miR-18a-3p, miR-26b-3p, miR-3605-5p, miR-548ad-5p, miR-424-3p, miR-382-3p, miR-642a-3p, miR-941, miR-550a-5p, miR-3177-3p, miR-450a-5p, miR-130a-3p, miR-324-5p, miR-5010-5p, miR-4433a-5p, miR-889-3p, miR-1284, miR-98-3p, miR-3940-3p, miR-10527-5p, miR-589-5p, miR-505-5p, miR-874-3p, miR-99b-3p, miR-381-3p	RNA sequencing	ND	[2]
					Ischemic cardiomyopathy controls vs. Healthy controls	miR-92a-3p, miR-27b-3p, miR-451a, miR-221-3p, miR-375-3p, let-7g-5p, miR-4732-5p, let-7d-5p, miR-30c-5p, miR-148b-3p, let-7f-5p, let-7b-5p, miR-126-3p, miR-16-2-3p, miR-10b-5p, miR-139-5p, miR-151a-3p, miR-26b-5p, miR-15a-5p, miR-185-5p, miR-98-5p, let-7a-5p, miR-126-5p,			

						miR-23b-3p, miR-7-5p, miR-342-3p, miR-132-3p, miR-335-3p, let-7c-5p, miR-191-5p, miR-29c-3p, miR-1260b, miR-125a-5p, miR-4732-3p, miR-365a-3p, miR-365b-3p, let-7e-5p, miR-576-5p, miR-425-3p, miR-100-5p, miR-409-3p, miR-320a-3p, miR-4685-3p, miR-30b-5p, miR-1301-3p, miR-320e, miR-654-3p, miR-532-5p, miR-196b-5p, miR-323b-3p, miR-7976, miR-625-3p, miR-483-5p, miR-485-3p, miR-421, miR-382-3p, miR-6734-5p, miR-324-5p, miR-1908-5p, miR-1290, miR-130a-3p, miR-1-3p, miR-28-5p, miR-145-3p, miR-3960, miR-642a-3p, miR-6815-5p, miR-889-3p, miR-432-5p, miR-1284, miR-381-3p			
					Chagas non-cardiomyopathy controls vs. Chagas cardiomyopathy cases	miR-629-5p, miR-18a-3p, miR-130b-3p, miR-5010-5p, miR-379-5p, miR-550a-3-5p, miR-628-5p, miR-1304-3p, miR-4485-3p, miR-1247-5p, miR-651-5p, miR-95-3p, miR-885-3p, miR-1228-5p, miR-483-3p, miR-150-3p, miR-495-3p, miR-139-5p, miR-511-5p, miR-885-5p, miR-125b-5p, miR-487b-3p, miR-27b-3p			
					Chagas non-cardiomyopathy controls vs. Ischemic cardiomyopathy controls	miR-941, miR-15b-3p, miR-361-3p, miR-92a-3p, miR-126-3p, let-7f-5p, miR-26a-5p, let-7a-5p, miR-16-2-3p, let-7b-5p, miR-98-5p, miR-23b-3p, miR-148b-3p, miR-432-5p, miR-382-5p, miR-151a-5p, miR-4732-3p, miR-221-3p, miR-323b-3p, miR-487b-3p, miR-625-3p, miR-374b-5p, miR-130b-3p, miR-654-3p, miR-191-5p, miR-192-5p, miR-10b-5p, miR-151a-3p, miR-150-5p, miR-329-3p, miR-1290, miR-339-5p, miR-328-3p, miR-574-5p, miR-30c-5p, miR-494-3p, miR-1260a, miR-320c, miR-342-3p, miR-125a-5p, miR-375-3p, miR-122-5p, let-7e-5p, miR-132-3p, miR-493-5p, miR-27b-3p, miR-4662a-5p, miR-4433b-5p, miR-342-5p, miR-99a-5p, miR-2110, miR-28-5p, miR-409-3p, miR-370-3p, miR-576-5p, miR-1307-3p, miR-320a-3p, miR-1260b, miR-885-5p, miR-145-5p, miR-3960, miR-942-5p, miR-19a-3p, miR-7847-3p, miR-125b-5p, miR-744-5p, miR-1301-3p, miR-6511a-3p, miR-145-3p, miR-4516, miR-1306-5p, miR-1-3p, miR-320b, miR-29c-3p, miR-532-3p, miR-501-3p, miR-483-3p, miR-139-5p, miR-150-3p, miR-483-5p, miR-532-5p, miR-23a-5p, miR-206, miR-6734-5p, miR-885-3p			
					Chagas cardiomyopathy cases vs. Ischemic cardiomyopathy controls	miR-495-3p, miR-191-3p, miR-16-2-3p, miR-7-5p, miR-151a-3p, miR-423-5p, miR-144-5p, miR-106b-3p, miR-148b-3p, miR-28-5p, miR-15a-5p, miR-501-3p, miR-320a-3p, miR-132-3p, miR-151b, miR-432-5p, miR-30b-5p, miR-4732-3p, miR-320d, miR-18a-3p, miR-19b-3p, miR-409-3p, miR-152-3p, miR-339-5p, miR-320e, miR-7847-3p, miR-1290, miR-576-5p, miR-483-5p, miR-4685-3p, miR-654-3p, miR-29c-3p, miR-4508, miR-1304-3p, miR-379-5p, miR-628-5p, miR-3960, miR-4485-3p, miR-431-5p, miR-95-3p, miR-130b-3p			
	Trypanosoma cruzi	Chagas disease	*In vivo*	Human left ventricular free wall heart tissue	CCC vs. Controls	miR-146a-5p, miR-155-5p, miR-193a-5p, miR-409-3p, miR-24-3p, miR-93-5p, miR-92a-3p, miR-192-5p, miR-127-3p, miR-30c-5p, miR-26a-5p, miR-532-5p, miR-376c-3p, miR-486-5p, miR-25-3p, miR-95-3p, miR-152-3p, miR-24-2-5p, miR-15b-5p, miR-378, miR-20a-3p, miR-744-5p, miR-99a-3p, miR-345-5p, miR-328-3p, miR-103a-3p, miR-33a-3p,	RT-qPCR on TLDA (TaqMan Low Density Array) microfluidic cards	ND	[3]

						miR-15a-5p, miR-423-5p, miR-411-5p, miR-28-5p, miR-296-5p, miR-335-5p, miR-29a-3p, miR-125b-5p, miR-340-5p, miR-20b-5p, miR-302b-3p, miR-151a-5p, miR-133b, miR-106b-5p, miR-379-5p, miR-133a-3p, miR-302d-3p, miR-19b-1-5p, miR-642a-5p, miR-9-3p, miR-145-3p, miR-1233-3p, miR-487b-3p, miR-185-5p, miR-212-3p, miR-22-5p, miR-422a, miR-376a-3p, miR-148a-3p, miR-365a-3p, miR-1-3p, miR-324-5p, miR-9-5p, miR-99a-5p, miR-101-3p, miR-361-5p, miR-27a-3p, miR-455-5p, miR-145-5p, miR-29c-3p, miR-378a-5p, miR-23b-3p, miR-224-5p, miR-455-3p, miR-203a-3p, miR-494-3p, miR-143-3p, miR-451a, miR-499a-5p, miR-27b-3p, miR-221-3p, miR-22-3p, miR-151a-3p			
**Virus**	Crimean-Congo haemorrhagic fever virus	Crimean-Congo haemorrhagic fever	*In vivo*	Whole blood	Non-fatal cases vs. Controls	miR-1268a, miR-3940-5p, miR-937-5p, miR-1249-3p, miR-296-5p, miR-144-3p, miR-144-5p, miR-148a-3p, miR-101-3p, miR-126-3p, miR-96-5p, miR-18a-5p, miR-27a-3p, miR-590-5p, miR-18b-5p	Microarray analysis	RT-qPCR	[4]
					Fatal cases vs. Controls	miR-4793-3p, miR-195-3p, miR-4440, miR-1909-5p, miR-548 am-5p, miR-7850-5p, miR-1908-3p, miR-6775-3p, miR-595, miR-7108-3p, let-7f-5p, let-7a-5p, let-7g-5p, miR-144-3p, miR-142-5p, miR-16-5p, miR-144-5p, miR-20b-5p, let-7d-5p, miR-15b-5p, miR-7975, miR-363-3p, miR-101-3p, miR-130a-3p, miR-103a-3p, miR-223-3p, miR-532-5p, miR-107, miR-126-3p, miR-106b-5p, miR-15a-5p, miR-148a-3p, let-7c-5p, miR-21-5p, miR-19b-3p, miR-16-2-3p, miR-550a-3-5p, miR-26b-5p, miR-185-5p, miR-625-5p, miR-7-5p, miR-150-5p, miR-29c-3p, miR-19a-3p, miR-20a-5p, miR-17-5p, miR-24-3p, miR-23a-3p, let-7i-5p, miR-96-5p, miR-23b-3p, miR-93-5p, miR-183-5p, miR-130b-3p, miR-532-3p, miR-590-5p, miR-502-3p, miR-26a-5p, miR-660-5p, let-7b-5p, miR-151a-5p, miR-7977, miR-18b-5p, miR-6073, miR-29a-3p, miR-500a-3p, miR-378i, miR-181a-5p, miR-30c-5p, miR-18a-5p, miR-27a-3p, miR-210-3p, miR-151b, miR-29b-3p, miR-505-5p			
					Fatal cases vs. Non-fatal cases	miR-1909-5p, miR-4767, miR-548 am-5p, miR-6511a-3p, miR-4785, miR-6775-3p, miR-7850-5p, miR-6784-3p, miR-4793-3p, miR-4440, miR-195-3p, miR-1910-5p, miR-3935, miR-1470, miR-6815-3p, miR-7975, miR-142-5p, miR-532-3p, let-7a-5p, miR-425-5p, miR-550a-3-5p, miR-19b-3p, let-7f-5p, miR-223-3p, miR-185-5p, miR-150-5p, miR-3653-3p, miR-16-5p, miR-25-3p, let-7c-5p, let-7g-5p, let-7d-5p, miR-30b-5p, miR-502-3p, miR-22-3p, miR-30c-5p, miR-361-3p, miR-140-3p, miR-103a-3p, miR-4732-5p, miR-331-3, miR-625-5p, miR-342-5p, miR-320e, miR-423-5p, miR-107, miR-15b-5p, miR-7977, miR-106b-5p, miR-500a-3p, miR-451a, miR-324-5p, miR-130b-3p, miR-24-3p, miR-181a-5p, miR-378i, miR-151a-5p, miR-192-5p, miR-7641, miR-652-3p, miR-320d, let-7b-5p, miR-550a-3p, miR-151b, miR-4306, miR-30d-5p			
	Dengue virus type 1 (DENV-1)	Dengue viral infection	*In vivo*	Whole blood	Cases vs. Controls	miR-1972, miR-518a-3p, miR-1538, miR-300, miR-21-5p, miR-589-5p, miR-1537-3p, miR-340-5p, miR-1207-5p, miR-	miRNA PCR arrays	RT-qPCR	[5]

						632, miR-934, miR-1227-3p, miR-152-3p, miR-596, miR-149-3p, miR-629-5p, miR-188-5p, miR-23a-5p, miR-590-5p, let-7g-3p, miR-421, miR-424-5p, miR-335-5p, miR-548a-3p, miR-320d, miR-1203, miR-148b-3p, miR-22-5p, miR-338-5p, miR-1271-5p, miR-497-5p, miR-551a, miR-362-3p, miR-223-5p, miR-708-3p, miR-339-3p, miR-425-3p, miR-877-5p, miR-28-3p, let-7d-3p, miR-185-3p, miR-200a-3p, miR-375, miR-370-3p, miR-20b-5p, -let-7b-5p, miR-365a-3p, miR-885-5p, miR-942-5p, miR-146a-5p, miR-379-5p, miR-769-5p, miR-409-3p			
	Zika virus (ZIKV strain Uganda 976 and strain PF13/251013-18)	Zika virus disease	*In vitro*	Long-term self-renewing neuroepithelial-like stem cells	-	miR-6786-5p, miR-6090 , miR-6746-3p miR-5690, miR-6075, miR-718, miR-6084 , miR-3195, miR-4667-5p, miR-6126, miR-3659, miR-4763-5p, miR-3621, miR-211-5p, miR-4634, miR-887-3p, miR-4800-5p, miR-638, miR-7161-3p, miR-4787-5p, miR-186-3p, miR-3614-3p, miR-4459, miR-6088, miR-6087, miR-4662a-5p, miR-3692-5p, miR-4449, miR-4516, miR-6509-5p, miR-6724-5p, miR-1181, miR-3960, miR-611, miR-3656, miR-1343-5p, miR-3141, miR-4792, miR-5787, miR-4743-5p, miR-6836-3p, miR-4654, miR-6510-5p, miR-6089, miR-759, miR-874-5p, miR-6836-5p, miR-6873-3p, miR-4800-3p, miR-1469, miR-6793-5p, miR-875-5p, miR-6876-3p, miR-4508, miR-6861-5p, miR-663a, miR-6870-5p, miR-6790-3p, miR-5585-5p, miR-4739, miR-4497, miR-183-5p, miR-7641, miR-4771, miR-589-5p, miR-92a-1-5p, miR-1261, miR-125b-1-3p, miR-7704, miR-182-5p	RNA sequencing	RT-qPCR	[6]

Note: Statistically significantly upregulated miRNAs are marked in red and statistically significantly downregulated miRNAs are marked in blue.

References

1. Geraci, N.S.; Tan, J.C.; McDowell, M.A. Characterization of microRNA expression profiles in Leishmania-infected human phagocytes. *Parasite Immunology* **2015**, *37*, 43-51, doi:https://doi.org/10.1111/pim.12156.
2. Antonietti, L.; Mariani, J.; Martínez, M.J.; Santalla, M.; Vensentini, N.; Kyle, D.A.; de Abreu, M.; Tajer, C.; Lacunza, E.; Ferrero, P. Circulating microRNAs as biomarkers of Chagas cardiomyopathy. *Frontiers in Cardiovascular Medicine* **2023**, *Volume 10 - 2023*, doi:10.3389/fcvm.2023.1250029.
3. Laugier, L.; Ferreira, L.R.P.; Ferreira, F.M.; Cabantous, S.; Frade, A.F.; Nunes, J.P.; Ribeiro, R.A.; Brochet, P.; Teixeira, P.C.; Santos, R.H.B.; et al. miRNAs may play a major role in the control of gene expression in key pathobiological processes in Chagas disease cardiomyopathy. *PLoS Negl Trop Dis* **2020**, *14*, e0008889, doi:10.1371/journal.pntd.0008889.
4. Arslan, S.; Engin, A.; Aydemir, E.I.; Sahin, N.O.; Bayyurt, B.; Sari, I.; Cosgun, Y.; Bakir, M. Identification of potential microRNA markers related to Crimean-Congo hemorrhagic fever disease. *J Cell Biochem* **2019**, *120*, 15506-15517, doi:10.1002/jcb.28817.
5. Ouyang, X.; Jiang, X.; Gu, D.; Zhang, Y.; Kong, S.K.; Jiang, C.; Xie, W. Dysregulated Serum MiRNA Profile and Promising Biomarkers in Dengue-infected Patients. *Int J Med Sci* **2016**, *13*, 195-205, doi:10.7150/ijms.13996.
6. Tabari, D.; Scholl, C.; Steffens, M.; Weickhardt, S.; Elgner, F.; Bender, D.; Herrlein, M.L.; Sabino, C.; Semkova, V.; Peitz, M.; et al. Impact of Zika Virus Infection on Human Neural Stem Cell MicroRNA Signatures. *Viruses* **2020**, *12*, doi:10.3390/v12111219.
