# Supplementary material for: MicroRNA Responses Associated with Vector-Borne Diseases in Humans and Arthropod Vectors: A Systematic Review
Source: Int J Mol Sci. 2026 Sep 22;27(19):8444. doi: 10.3390/ijms27198444 (PMC13664826; doi:10.3390/ijms27198444)
Supplement: Supplementary file 1 [file ijms-27-08444-s001.zip › Supplementary Tables S5 & S6. Data Vectors Other.pdf]

Supplementary Table S5. Differentially expressed miRNAs in vectors associated with VBDs.

Ectoparasite Type	Species	Sample type Tissue/organ/specimen/ database	Most common differentially expressed miRNAs	References
**Tick**	*Haemaphysalis longicornis*	Saliva	hlo-miR-100_2, hlo-miR-315, hlo-miR-184_1, hlo-miR-100-5p_2, hlo-miR-5307, hlo-miR-184-3p_3, hlo-let-7-5p_6, hlo-miR-71_5, hlo-miR-1-3p_6, hlo-miR-10-5p_2	[1]
		Salivary gland	hlo-miR-1, hlo-miR-375, hlo-miR-184, hlo-miR-263a, hlo-bantam, hlo-miR-739, hlo-miR-317, hlo-let-7, hlo-miR-275, hlo-miR-183	
	Hyalomma asiaticum	Midgut of unfed adult tick	has-nov-miR-17#	[2]
	Haemaphysalis longicornis	Salivary gland, midgut, ovary, and Malpighian tubules)	hlo-miR-2#	[3]
	Haemaphysalis longicornis	Eggs	hlo-miR-315, hlo-miR-10, hlo-miR-279, hlo-miR-1 and hlo-miR-71	[4]
		Unfed larvae	hlo-miR-184, hlo-miR-276, hlo-miR-1, hlo-miR-10, hlo-miR-285, hlo-hlo-mir-1#, hlo-hlomir-2#, hlo-mir-3#	
		Unfed nymphs	hlo-miR-79, hlo-miR-277a, hlo-miR-219b, hlo-miR-125, hlo-miR-210, hlo-miR-2001, hlo-miR-87, hlo-miR-133, hlo-miR-184, hlo-mir-1#, hlomir-2#, hlo-mir-3#, hlo-miR-9a, hlo-miR-bantam	
		Unfed adults	hlo-miR-5316b, hlo-miR-210, hlo-miR-125, hlo-miR-252, hlo-miR-100, hlo-miR-219, hlo-miR-79, hlo-miR-219a, hlo-miR-5308, hlo-miR-79, hlo-mir-1#, hlo-mir-2#, hlo-mir-3#, hlo-mir-8#, hlo-miR-1, hlo-miR-100, hlo-miR-750, hlo-miR-bantam, hlo-miR-10	
	Haemaphysalis longicornis	*H. longicornis* wild-type population	hlo-miR-1-3p, hlo-miR-29-5p and hlo-let-7-5p	[5]
		H. longicornis cultured population	hlo-miR-1-3p, hlo-miR-1, hlo-miR-29-5p and hlo-let-7-5p	
	Hyalomma anatolicum anatolicum	unfed-adult female ticks	haa-miR-001#, haa-miR-002#,haa-miR-003#,haa-miR-004#,haa-miR-005#,haa-miR-006#,haa-miR-007#,haa-miR-008#,haa-miR-009#,haa-miR-010#,haa-miR-011#,haa-miR-012#,haa-miR-013#,haa-miR-014#,haa-miR-015#,haa-miR-016#,haa-miR-017#,haa-miR-018#,haa-miR-019#,haa-miR-020#,haa-miR-021#,haa-miR-022#,haa-miR-023#,haa-miR-024#,haa-miR-025#,haa-miR-026#,haa-miR-027#,haa-miR-028#,haa-miR-029#	[6]
	Haemaphysalis longicornis	Fed adults, midgut	hlo-miR-275	[7]
	Hyalomma asiaticum	Partially engorged adult female ticks	has-let-7	[8]
	Haemaphysalis longicornis	Adult tick saliva	hlo-miR-71, hlo-miR-184, hlo-miR-2b, hlo-miR-5312, hlo-miR-2001, hlo-miR-2a, hlo-miR-1, hlo-miR-285, hlo-miR-96, hlo-miR-315, hlo-miR-315, hlo-miR-275, hlo-miR-8, hlo-miR-1, hlo-miR-276, hlo-miR-750, hlo-let-7, hlo-miR-2a, hlo-miR-279	[9]
	Rhipicephalus microplus	Eggs	rmi-miR-4175-3p, rmi-miR-4419b, rmi-miR-5187-5p, rmi-miR-483-3p, rmi-miR-1421, rmi-m0005#, m0014#	[10]
		Larvae	rmi-miR-5187-5p, rmi-miR-2371, rmi-miR-2126, rmi-miR-4908-3p, rmi-miR-669a-3-3p, rmi-miR-1183, rmi-m00018#, rmi-m0004#, rmi-m0005#	
		Female adults	rmi-miR-4175-3p, rmi-miR-4419b, rmi-miR-2371, rmi-miR-2126, rmi-miR-516-3p, rmi-miR-26b-5p, rmi-m00018#, rmi-m0004#, rmi-m0014#	
	Haemaphysalis longicornis	Salivary glands	miR-1, miR-375, miR-184, miR-263a, Bantam, miR-1268, miR-276a, miR-276a, miR-739, miR-184b, mir-1810, mir-2138, mir-2140, mir-425#, mir-429, mir-516, miR-10-5p, miR-1183, miR-125a-3p, miR-1293, miR-1-3p, miR-1538, miR-1591, miR-2126, miR-25, miR-2579, miR-260, miR-279a, miR-2941-1, miR-2973, miR-311, miR-3130-3p, miR-375-3p, miR-4000i-5p, miR-4001a-5p, miR-4006b-5p, miR4078-5p	[11]
	Ixodes ricinus	Adult salivary gland	iri-mir-X37-5p, iri-miR-252b-3p, iri-miR-100-5p, iri-miR-1175-3p, iri-miR-125b-5p, iri-miR-71-5p, iri-miR-278-5p, iri-miR-263a-5p, iri-miR-125b-3p, iri-mir-X14-3p, iri-mir-X7-3p, iri-mir-X11-5p, iri-miR-375-5p, iri-miR-10a-5p, iri-miR-2001-3p, iri-miR-X21-3p, iri-miR-X1f-5p, iri-miR-X1b-3p, iri-miR-275-5p, iri-mir-X31-5p, iri-mir-X33-5p, iri-miR-275-3p, iri-miR-2b2-3p, iri-miR-3931-5p, iri-miR-92-3p, iri-mir-X4-3p, iri-miR-375-3p, iri-mir-X24-3p, iri-miR-252b-5p, iri-miR-3931-3p, iri-mir-X2-3p	[12]
		Nymphal salivary gland	iri-mir-X2-3p, iri-miR-9a-5p, iri-miR-X1a-5p, iri-miR-X1b-5p, iri-miR-X1c-5p, iri-miR-34-5p, iri-miR-1-3p, iri-miR-X1h-5p	

		Nymphs	iri-miR-375- 3p, iri-miR-92-3p, iri-mir-X18-5p, and iri-miR-252b-5p, iri-miR-34-5p	
		Adults	iri-miR-375- 3p, iri-miR-92-3p, iri-mir-X18-5p, and iri-miR-252b-5p, iri-miR-263a-5p, iri-miR-375-5p	
	Rhipicephalus sanguineus	Adult males	rsa-miR-m0001-5p#, rsa-miR-m0001-3p#, rsa-miR-m00012-5p#, rsa-miR-m00012-3p#, rsa-miR-m00028-5p#, rsa-miR-m00028-3p#	[13]
		Adult females	rsa-miR-m0006-5p#, rsa-miR-m0009-5p#, rsa-miR-m00010-5p#, rsa-miR-m00010-3p#	
**Louse**	*Pediculus humanus*	1st instar nymphs	bantam, let-7, mir-1, mir-10, mir-1000, mir-12, mir-124, mir-125, mir-125as, mir-133, mir-137, mir-14, mir-184, mir-190, mir-2, mir-2, mir-2, mir-2, mir-2, mir-210, mir-219, mir-25, mir-25, mir-252, mir-263, mir-263, mir-275, mir-276, mir-277, mir-278, mir-279, mir-305, mir-31, mir-316, mir-317, mir-34, mir-375, mir-46, mir-67, mir-7, mir-71, mir-8, mir-87, mir-9, mir-9, mir-9, mir-9, mir-9, mir-927, mir-929, mir-965, mir-981, mir-99, mir-993, mir-995, mir-iab-4, mir-iab-4as	[14]
**Mite**	*Sarcoptes scabiei*	Starved mites, embedded mites with infected host skin, uninfected host skin	miR-1175-5p, miR-276, miR-71-5p, miR-190, miR-146a-5p, miR21-5p, miR-181-5p	[15]
**Tsetse Fly**	*Glossina morsitans*	miRBase (www.mirbase.org)	gmr-miR-619-5p, gmr-miR-466i-5p, gmr-miR-487a-5p, gmr-miR-4057-3p, gmr-miR-9700b-3p, gmr-let-7-5p, gmr-let-7, gmr-let-7-3p, gmr-let-7a-5p, gmr-let-7a, gmr-miR-6040-3p, gmr-miR-2756-3p, gmr-miR-450a-3p, gmr-miR-3721-3p, gmr-miR-10454c-3p, gmr-miR-21-3p, gmr-miR-13a, gmr-miR-13a-3p, gmr-miR-881-5p, gmr-miR-975-3p, gmr-miR-3051, gmr-miR-2033-5p, gmr-miR-3820-5p, gmr-miR-3851a-3p, gmr-miR-10486-3p, gmr-miR-2490-3p, gmr-miR-100-3p, gmr-miR-203a-3p, gmr-miR-4979-3p, gmr-miR-9865-5p, gmr-miR-15a-5p, gmr-miR-100-5p, gmr-miR-1926, gmr-miR-2491-3p, gmr-miR-23a-5p	[16]

Note: Significantly upregulated miRNAs are marked in red, significantly downregulated miRNAs are marked in blue and novel miRNAs are marked with #.

Supplementary Table S6. Differentially expressed miRNAs identified in various other in-vitro host cell models.

Host Type	Cell type	Infectious Agent (IA) Characteristics			Most common differentially expressed miRNAs	miRNA profiling technique	Validated by	Reference
		Species	IA Type	VBD				
**Human and Mosquito**	*Aedes aegypti* 2 cell line & HEK-293T cell line	Chikungunya virus (CHIKV)	Virus	Chikungunya	miR-2944b-5p	qRT-PCR	ND	[17]
**Mosquito and Other**	C6/36 cells (*Aedes albopictus* clone C6/36) and vero cells	dengue virus type 2	Virus	Dengue	miR-184, miR-451, miR-1-3p, miR-486-3p, miR-486-5p, miR-162, miR-614, miR-378d, miR-122a-5p, miR-32-5p, miR-34c-5p, miR-21-3p, miR-97, miR-29b-3p, miR-99a-5p, miR-143-3p, miR-30a-5p, miR-30e-5p, miR-424-5p	RNA sequencing	qRT-PCR	[18]
	Mosquito C6/36 HT cell line and Vero cells	Dengue (DENV-1 (strain Bga-07), DENV-2 (strain 109–05) and DENV-4 (strain Bga-06)	Virus	Dengue	miR-133a	qRT-PCR	ND	[19]
**Other**	PK-15 cells	Japanese encephalitis	Virus	Japanese encephalitis	miR-21, let-7f, miR-30a-5p, miR-100, miR-29a, miR-152, miR-10a-5p, miR-19b , miR-26a, miR-182, miR-10a-3p, miR-10b, miR-101, miR-126-5p, miR-142-5p, miR-155-5p, miR-17-3p, miR-18a, miR-19a, miR-181a, miR-196b, miR-221-3p, miR-98, let-7d-3p, let-7d-5p	RNA sequencing	qRT-PCR	[20]
	BV-2 cells (mouse microglia cells)	Japanese encephalitis	Virus	Japanese encephalitis	miR-155	qRT-PCR	ND	[21]
	Mouse fibroblast L cells	chikungunya virus (CHIKV)	Virus	Chikungunya	miR-28a-3p, miR-30c-1-3p, miR-376c-3p, miR-135b-5p, miR-466b-3p, miR-486a, miR-350-3p, miR-30d-3p, miR-194-1, miR-149-5p, miR-214-3p, miR-27b-5p, miR-93-3p, miR-467e-5p, miR-292a-3p, miR-7b-5p, miR-29b-1-5p, miR-132-3p, miR-466a-3p, miR-7a-1-3p, miR-546, miR-381-3p, miR-15b-3p, miR-22-5p, let-7f-5p, miR-352, miR-423-5p, miR-186-3p, miR-339-3p, miR-684, miR-375-3p, miR-351-5p, miR-378a-5p, let-7c-3p, miR-193b-3p, miR-331-3p, miR-18a-3p, miR-191-3p, miR-17-3p, miR-296-5p, miR-126-3p, miR-671-3p, miR-129-3p, miR-30b-3p, miR-741-3p, miR-27a-5p, miR-467b-3p, miR-381-3p, miR-878-3p, miR-150-5p, miR-328-3p, miR-704, miR-99b-3p, miR-425-5p, miR-294-3p, miR-451a, miR-295-3p, miR-582-3p, miR-465a-3p, miR-181a-5p, miR-20a-3p, miR-877-3p, let-7b-5p, let-7a-5p, miR-463-5p, miR-92a-3p, miR-877-5p, miR-135a-1-3p, miR-200a-3p, miR-664-3p, miR-181c-5p, miR-883b-3p, miR-139-5p, miR-378a-3p, miR-409-3p	Low density microarray	qRT-PCR	[22]
	Bone marrow-derived macrophages (BMDMs) and mouse embryonic fibroblasts (MEFs)	West Nile virus	Virus	West Nile virus	miR-155	qRT-PCR	ND	[23]

Note: Significantly upregulated miRNAs are marked in red, significantly downregulated miRNAs are marked in blue and novel miRNAs are marked with #.

References

1. Malik, M.I.; Nawaz, M.; Hassan, I.A.; Zhang, H.; Gong, H.; Cao, J.; Zhou, Y.; Zhou, J. A microRNA profile of saliva and the role of miR-375 in Haemaphysalis longicornis (Ixodida: Ixodidae). *Parasites and Vectors* **2019**, *12*, (1 February 2019), doi:10.1186/s13071-019-3318-x.
2. Luo, J.; Wu, F.; Liu, W.; Ren, Q.; Diao, P.; Guan, G.; Luo, J.; Yin, H.; Liu, G. A Novel MicroRNA and the Target Gene TAB2 Can Regulate the Process of Sucking Blood in and the Spawn Rate of Hyalomma asiaticum (Acari: Ixodidae) Ticks. *Front Immunol* **2022**, *13*, 930532, doi:10.3389/fimmu.2022.930532.
3. Liu, W.G.; Luo, J.; Ren, Q.Y.; Qu, Z.Q.; Lin, H.L.; Xu, X.F.; Ni, J.; Xiao, R.H.; Chen, R.G.; Rashid, M.; et al. A Novel miRNA-hlo-miR-2-Serves as a Regulatory Factor That Controls Molting Events by Targeting CPR1 in Haemaphysalis longicornis Nymphs. *Front Microbiol* **2020**, *11*, 1098, doi:10.3389/fmicb.2020.01098.
4. Liu, W.G.; Guo, J.H.; Luo, J.; Ren, Q.Y.; Chen, Z.; Qu, Z.Q.; Wu, Z.G.; Ni, J.; Xu, X.F.; Rashid, M.; et al. Analysis of microRNA expression profiles dynamic in different life stages of Haemaphysalis longicornis ticks by deep sequencing of small RNA libraries. *TICKS AND TICK-BORNE DISEASES* **2020**, *11*, doi:10.1016/j.ttbdis.2020.101427.
5. Luo, J.; Ren, Q.; Chen, Z.; Liu, W.; Qu, Z.; Xiao, R.; Chen, R.; Lin, H.; Wu, Z.; Luo, J.; et al. Comparative analysis of microRNA profiles between wild and cultured Haemaphysalis longicornis (Acari, Ixodidae) ticks. *Parasite* **2019**, *26*, 18, doi:10.1051/parasite/2019018.
6. Luo, J.; Liu, G.Y.; Chen, Z.; Ren, Q.Y.; Yin, H.; Luo, J.X.; Wang, H. Identification and characterization of microRNAs by deep-sequencing in Hyalomma anatolicum anatolicum (Acari: Ixodidae) ticks. *Gene* **2015**, *564*, 125-133, doi:10.1016/j.gene.2015.01.019.
7. Hao, J.; Luo, J.; Chen, Z.; Ren, Q.; Guo, J.; Liu, X.; Chen, Q.; Wu, F.; Wang, Z.; Luo, J.; et al. MicroRNA-275 and its target Vitellogenin-2 are crucial in ovary development and blood digestion of Haemaphysalis longicornis. *Parasit Vectors* **2017**, *10*, 253, doi:10.1186/s13071-017-2153-1.
8. Wu, F.; Luo, J.; Chen, Z.; Ren, Q.; Xiao, R.; Liu, W.; Hao, J.; Liu, X.; Guo, J.; Qu, Z.; et al. MicroRNA let-7 regulates the expression of ecdysteroid receptor (ECR) in Hyalomma asiaticum (Acari: Ixodidae) ticks. *Parasit Vectors* **2019**, *12*, 235, doi:10.1186/s13071-019-3488-6.
9. Nawaz, M.; Malik, M.I.; Zhang, H.S.; Gebremedhin, M.B.; Cao, J.; Zhou, Y.Z.; Zhou, J.L. miRNA profile of extracellular vesicles isolated from saliva of Haemaphysalis longicornis tick. *ACTA TROPICA* **2020**, *212*, doi:10.1016/j.actatropica.2020.105718.
10. Luo, J.; Zhao, S.; Ren, Q.; Wang, Q.; Chen, Z.; Cui, J.; Jing, Y.; Liu, P.; Yan, R.; Song, X.; et al. Dynamic Analysis of microRNAs from Different Life Stages of Rhipicephalus microplus (Acari: Ixodidae) by High-Throughput Sequencing. *Pathogens* **2022**, *11*, doi:10.3390/pathogens11101148.
11. Zhou, J.L.; Zhou, Y.Z.; Cao, J.; Zhang, H.S.; Yu, Y.F. Distinctive microRNA profiles in the salivary glands of Haemaphysalis longicornis related to tick blood-feeding. *EXPERIMENTAL AND APPLIED ACAROLOGY* **2013**, *59*, 339-349, doi:10.1007/s10493-012-9604-3.
12. Hackenberg, M.; Langenberger, D.; Schwarz, A.; Erhart, J.; Kotsyfakis, M. In silico target network analysis of de novo-discovered, tick saliva-specific microRNAs reveals important combinatorial effects in their interference with vertebrate host physiology. *RNA* **2017**, *23*, 1259-1269, doi:10.1261/rna.061168.117.
13. Shao, C.C.; Xu, M.J.; Chen, Y.Z.; Tao, J.P.; Zhu, X.Q. Comparative Profiling of MicroRNAs in Male and Female Rhipicephalus sanguineus. *Appl Biochem Biotechnol* **2015**, *176*, 1928-1936, doi:10.1007/s12010-015-1688-x.
14. Kirkness, E.F.; Haas, B.J.; Sun, W.L.; Braig, H.R.; Perotti, M.A.; Clark, J.M.; Lee, S.H.; Robertson, H.M.; Kennedy, R.C.; Elhaik, E.; et al. Genome sequences of the human body louse and its primary endosymbiont provide insights into the permanent parasitic lifestyle. *PROCEEDINGS OF THE NATIONAL ACADEMY OF SCIENCES OF THE UNITED STATES OF AMERICA* **2010**, *107*, 12168-12173, doi:10.1073/pnas.1003379107.
15. He, R.; Gu, X.; Lai, W.; Peng, X.; Yang, G. Transcriptome-microRNA analysis of Sarcoptes scabiei and host immune response. *PLoS One* **2017**, *12*, e0177733, doi:10.1371/journal.pone.0177733.
16. Yang, Z.; Wang, M.; Zeng, X.; Wan, A.T.; Tsui, S.K. In silico analysis of proteins and microRNAs related to human African trypanosomiasis in tsetse fly. *Comput Biol Chem* **2020**, *88*, 107347, doi:10.1016/j.compbiolchem.2020.107347.
17. Dubey, S.K.; Shrinet, J.; Sunil, S. Aedes aegypti microRNA, miR-2944b-5p interacts with 3'UTR of chikungunya virus and cellular target vps-13 to regulate viral replication. *PLoS Negl Trop Dis* **2019**, *13*, e0007429, doi:10.1371/journal.pntd.0007429.
18. Yang, J.J.; Lin, Y.; Jiang, L.M.; Xi, J.M.; Wang, X.D.; Guan, J.Q.; Chen, J.Y.; Pan, Y.; Luo, J.; Ye, C.; Sun, Q.M. Comparison analysis of microRNAs in response to dengue virus type 2 infection between the Vero cell-adapted strain and its source, the clinical C6/36 isolated strain. *VIRUS RESEARCH* **2018**, *250*, 65-74, doi:10.1016/j.virusres.2018.04.011.
19. Castillo, J.A.; Castrillón, J.C.; Diosa-Toro, M.; Betancur, J.G.; St Laurent, G., 3rd; Smit, J.M.; Urcuqui-Inchima, S. Complex interaction between dengue virus replication and expression of miRNA-133a. *BMC Infect Dis* **2016**, *16*, 29, doi:10.1186/s12879-016-1364-y.
20. Cai, Y.; Zhu, L.; Zhou, Y.; Liu, X.; Liu, X.; Li, X.; Lang, Q.; Qiao, X.; Xu, Z. Identification and analysis of differentially-expressed microRNAs in Japanese encephalitis virus-infected PK-15 cells with deep sequencing. *International Journal of Molecular Sciences* **2015**, *16*, 2204-2219.
21. Thounaojam, M.C.; Kiran, K.; Kaushik, D.K.; Shalini, S.; Anita, M.; Shankar, S.K.; Anirban, B. MicroRNA 155 regulates Japanese encephalitis virus-induced inflammatory response by targeting Src homology 2-containing inositol phosphatase 1. *Journal of Virology* **2014**, *88*, 4798-4810, doi:10.1128/JVI.02979-13.
22. Sharma, A.; Balakathiresan, N.S.; Maheshwari, R.K. Chikungunya virus infection alters expression of microRNAs involved in cellular proliferation, immune response and apoptosis. *Intervirology* **2015**, *58*, 332-341, doi:10.1159/000441309.
23. Natekar, J.P.; Rothan, H.A.; Arora, K.; Strate, P.G.; Kumar, M. Cellular microRNA-155 Regulates Virus-Induced Inflammatory Response and Protects against Lethal West Nile Virus Infection. *Viruses* **2019**, *12*, doi:10.3390/v12010009.
